# Genome-wide associations with longevity and reproductive traits in U.S. rangeland ewes

**DOI:** 10.3389/fgene.2024.1398123

**Published:** 2024-05-27

**Authors:** Jamin A. Smitchger, J. Bret Taylor, Michelle R. Mousel, Daniel Schaub, Jacob W. Thorne, Gabrielle M. Becker, Brenda M. Murdoch

**Affiliations:** ^1^ Department of Animal, Veterinary and Food Sciences, University of Idaho, Moscow, ID, United States; ^2^ USDA, Agriculture Research Service, Range Sheep Production Efficiency Research Unit, U.S. Sheep Experiment Station, Dubois, ID, United States; ^3^ Animal Diseases Research Unit, Agricultural Research Service, US Department of Agriculture, Pullman, WA, United States; ^4^ Texas A&M AgriLife Research and Extension, San Angelo, TX, United States

**Keywords:** GWAS, sheep, productive life, premature culling, lifetime lambs born, lifetime lambs weaned, litter size, weaning weight

## Abstract

**Introduction:** Improving ewe longevity is an important breeding and management goal, as death loss and early culling of mature ewes are economic burdens in the sheep industry. Ewe longevity can be improved by selecting for positive reproductive outcomes. However, the breeding approaches for accomplishing this come with the challenge of recording a lifetime trait. Characterizing genetic factors underpinning ewe longevity and related traits could result in the development of genomic selection strategies to improve the stayability of sheep through early, informed selection of replacement ewes.

**Methods:** Towards this aim, a genome-wide association study (GWAS) was performed to identify genetic markers associated with ewe longevity, reproductive, and production traits. Traits evaluated included longevity (i.e., length of time in the flock), parity and the lifetime number of lambs born, lambs born alive, lambs weaned, and weight of lambs weaned. Ewe records from previous studies were used. Specifically, Rambouillet (n = 480), Polypay (n = 404), Suffolk (n = 182), and Columbia (n = 64) breed ewes (N = 1,130) were analyzed against 503,617 SNPs in across-breed and within-breed GWAS conducted with the Bayesian-information and Linkage-disequilibrium Iteratively Nested Keyway (BLINK) model in R.

**Results:** The across-breed GWAS identified 25 significant SNPs and the within-breed GWAS for Rambouillet, Polypay, and Suffolk ewes identified an additional 19 significant SNPs. The most significant markers were rs411309094 (13:22,467,143) associated with longevity in across-breed GWAS (*p*-value = 8.3E-13) and rs429525276 (2:148,398,336) associated with both longevity (*p*-value = 6.4E-15) and parity (*p*-value = 4.8E-15) in Rambouillet GWAS. Significant SNPs were identified within or in proximity (±50 kb) of genes with known or proposed roles in reproduction, dentition, and the immune system. These genes include *ALPL, ANOS1, ARHGEF26, ASIC2, ASTN2, ATP8A2, CAMK2D, CEP89, DISC1, ITGB6, KCNH8, MBNL3, MINDY4, MTSS1, PLEKHA7, PRIM2, RNF43, ROBO2, SLCO1A2, TMEM266, TNFRSF21,* and *ZNF804B*.

**Discussion:** This study proposes multiple SNPs as candidates for use in selection indices and suggests genes for further research towards improving understanding of the genetic factors contributing to longevity, reproductive, and production traits of ewes.

## 1 Introduction

Extending the productive lifespan of ewes is one strategy for increasing economic return in sheep production. Longevity can be described as the length of an animal’s productive life in the flock (McLaren et al., 2020), and has previously been analyzed in sheep using age, lifetime performance, and average performance as indicator traits (Kern et al., 2010; Pettigrew et al., 2019). For most commercial sheep producers, the majority of annual profits comes through the sale of lambs for slaughter (Flay et al., 2022). Replacing ewes that died or were culled because their ability to be productive was compromised can reduce lamb sale revenue and add extra feed and management costs associated with developing replacements (Mekkawy et al., 2009; Abdelqader et al., 2012). Flocks with ewes that remain productive longer do not have to incur the year-over-year costs associated with mature ewe replacement. Additionally, flocks with a greater composition of mature ewes have increased production outputs, suggesting further economic incentive to improve longevity (Conington et al., 2004; Taylor et al., 2009; Farrell et al., 2019).

Longevity is a complex trait that can be influenced by many factors, such as management, reproductive, and animal health (Getachew et al., 2015; Watt et al., 2021). Management and reproductive traits such as the birth litter size of the ewe, age at first lambing, litter size, lambing interval, and number of lambings (parity) have been found to be associated with ewe longevity, to varying degrees (Getachew et al., 2015; Douhard et al., 2016; McLaren et al., 2020; Hanna et al., 2023). Culling and animal death can be impacted by environmental conditions such as extreme climates, predation, and/or disease (Getachew et al., 2015; Mabille et al., 2015), and susceptibility to dystocia and other common health concerns also contribute to animal loss (Farrell et al., 2019; Bruce et al., 2021).

Previous studies have suggested that genetics can influence longevity. Ewe stayability (*h* ^*2*^ = 0.04–0.11) and length of productive life (*h* ^*2*^ = 0.05–0.13) have been shown to be lowly heritable (Borg et al., 2009; Zishiri et al., 2013; Lee et al., 2015), indicating that these traits may respond to selection. Longevity cannot be calculated until the end of a ewe’s lifespan; therefore, selecting for genetic variants that influence longevity could be a pragmatic approach for making more rapid progress. Genome-wide association scan methods have been used to evaluate genetics associated with longevity traits in Mediterranean Chios sheep (Tsartsianidou et al., 2021). The previous study by Tsartsianidou et al. (2021) found a lack of genetic diversity associated with longevity traits in the sampled population. Therefore, there is a need for further research in this area with populations relevant to the United States (U.S.) sheep industry. Identifying makers associated with longevity would not only aid genomic selection efforts, but also improve the current understanding of the genes and biological mechanisms underpinning variation in longevity. Towards this aim, records of Rambouillet, Polypay, Suffolk, and Columbia breed ewes queried from previous projects were evaluated through genome-wide association study (GWAS) for longevity (number of years in the flock) and lifetime reproductive and production traits, including number of lambs born, number of lambs born alive, number of lambs weaned, and weight of lambs weaned.

## 2 Materials and Methods

### 2.1 Sheep management and records

The sheep used in this study were managed under an extensive rangeland production system at the United States Department of Agriculture (USDA), Agricultural Research Service (ARS), Range Sheep Production Efficiency Research Unit, U.S. Sheep Experiment Station (USSES) located near Dubois, Idaho. The USSES records from 1999 to 2021 were utilized. The study group of ewes originated from former projects when ewes were retained and/or evaluated beyond the standard culling age of 7 years. For the current dataset, ewes were required to be a minimum of 1.5 years of age and to have left the flock, through death or culling, prior to the conclusion of the record sampling period. Data on ewes greater than 10 years of age were not available. Accordingly, the dataset was biased towards a greater proportion of older ewes than would be expected to exist under standard management practices. In total, records of 1,130 ewes were utilized, including ewes of Rambouillet, Polypay, Suffolk, and Columbia USSES breed designation.

Longevity was calculated as the difference between the disposal date (sale or death) and birthdate for each ewe, reported in years. Ewes without definite sale or death dates were not included in this trait. For the purposes of GWAS, longevity was evaluated at two levels: ewes >1.5 years of age (Longevity_1.5_) and ewes >3.35 years of age (Longevity_3.35_) at the time of death/culling. The Longevity_1.5_ level included ewes that remained after initial culling decisions (N = 1,045; Rambouillet n = 433, Polypay n = 388, Suffolk n = 161, Columbia n = 63), while Longevity_3.35_ was comprised of ewes that had experienced at least two lambing seasons (N = 959; Rambouillet n = 412, Polypay n = 362, Suffolk n = 128, Columbia n = 57). The threshold of 3.35 years was selected from the distribution of longevity records. No ewes left the flock between 3.30 years and 3.39 years of age: ewes above this break in the data were carried forward as Longevity_3.35_.

As ewe longevity was expected to be influenced by performance, reproductive and production traits were evaluated as well. From the study group, lambing and weaning data were available for N = 1,130 ewes, including 480 Rambouillet, 404 Polypay, 182 Suffolk, and 64 Columbia sheep. Reproductive and production phenotypes were investigated as cumulative lifetime (LT) traits, being the sum of each ewe’s records over the entire lifespan in the flock. The traits included number of lambs born, number of lambs born alive, number of lambs weaned, and weight of lambs weaned (kg). Lifetime parity, or the total number of lambing per ewe, was also evaluated.

### 2.2 Statistical analyses of longevity, reproductive, and production traits

All phenotypic data were confirmed to be normally distributed through the Shapiro-Wilk test in R version 4.2.3 (R Core Team, 2021). Descriptive statistics, which included the arithmetic mean, standard deviation, minimum, and maximum value, were calculated for each trait. The directions and strengths of relationships between traits were evaluated through Pearson correlation testing. Since correlation testing required pairwise analyses of non-missing data, this analysis was restricted to the 1,045 ewes with non-missing data for longevity_1.5_, parity, and LT traits. The ‘corrplot’ package in R was used to compute Pearson correlation coefficients and *p*-values (Wei and Simko, 2021).

### 2.3 Preparation of genotype data

The methods relating to the collection of genotypic data have been previously described (Mousel et al., 2021). In brief, blood samples were collected from each ewe and DNA were extracted using either the Invitrogen GeneCatcher gDNA 3–10 mL Blood Kit (Life Technologies, Carlsbad, CA) or the Gentra PureGene (Qiagen, Germantown, MD). The DNA samples were provided to Geneseek Inc. (Lincoln, NE) for genotyping with the high-density (HD) Illumina 600 K SNP BeadChip (Illumina Inc., San Diego, CA, United States of America), comprised of 606,006 markers.

Genotype quality control was conducted using SNP and Variation Suite™ v8.9.1 (SNP, 2022). Markers were removed from analyses by call rate (CR) < 90.0 (21,055 SNPs) and Hardy-Weinberg Equilibrium *p*-value <1.0E-06 (47,953 SNPs). Additionally, markers with a minor allele frequency (MAF) < 0.01 (30,635 SNPs) were removed in accordance with standard quality control practices (Turner et al., 2011). Duplicate markers were filtered to retain the marker with the highest CR at each unique position (2,746 SNPs). All sheep had a sample CR of >0.90. In total, there were 1,130 sheep and 503,617 markers, including 481,813 autosomal and 21,804 X-chromosome markers, retained for analyses.

### 2.4 Genome-wide association studies

To understand genomic associations with longevity, reproductive, and production traits in this population of sheep, two levels of GWAS were conducted. First, an across-breed GWAS was conducted with all study animals. Following this GWAS approach, the Rambouillet, Polypay, and Suffolk breeds were evaluated in breed specific GWAS for the same traits. Due to the limited number, Columbia ewes were not analyzed in a breed specific GWAS.

The software Tassel 5 (Bradbury et al., 2007) was used to convert the genotype files to hapmap format for input into R. All GWAS were conducted using the multi-locus Bayesian-information and Linkage-disequilibrium Iteratively Nested Keyway (BLINK) model integrated in version 3 of the Genome Association and Prediction Integrated Tool (GAPIT) R Software package (Wang and Zhang, 2021). A principal component analysis (PCA) was conducted for across- and within-breed analyses and the first three principal components (PC) were used to correct for population stratification in each GWAS (Supplementary Figure S1). The proportion of variance explained (PVE) was calculated for the first three PCs as the eigenvalue divided by the sum of eigenvalues. The PVE of PC1, PC2, and PC3 were 34.8%, 28.7%, and 9.3%, respectively. All GWAS were investigated under additive inheritance models. The threshold for declaring genome-wide significance for each trait was determined by the Benjamini Hochberg FDR-adjusted *p*-value. In the across-breed GWAS, significance thresholds varied from -log10(*p*-value) ≥ 6.23 to ≥ 8.12. In the within-breed GWAS, the Rambouillet breed thresholds ranged from -log10(*p*-value) ≥ 6.55 to ≥ 10.12 and the Polypay thresholds ranged from -log10(*p*-value) ≥ 6.50 to ≥ 8.51. A single trait was significant in Suffolk GWAS, resulting in a threshold of -log10(*p*-value) ≥ 9.36. Due to the variation in FDR-adjusted *p*-value thresholds, the Bonferroni-adjusted *p*-value was used for visualization in all Manhattan plots. Effect sizes were estimated for each SNP to correspond to the allele that was further in alphabetical order. That is, for an A/G SNP, the effect size was estimated to correspond with the effect of the G allele, and for a T/C SNP, the effect size was estimated to correspond with the T allele. GWAS results were visualized with the package ‘CMplot’ in R (Yin, 2022). The linkage disequilibrium (LD) decay of each of the four breeds were analyzed with PopLDdecay under default parameters (Zhang et al., 2019).

### 2.5 Post hoc analysis of GWAS results

To understand whether sheep breed could be driving specific SNP associations within the dataset, analysis of variance (ANOVA) and Tukey-HSD tests were conducted in R using the package ‘rstatix’ and visualized with ‘ggplot2’ and ‘patchwork’ (Wickham, 2016; Pedersen, 2020; Kassambara, 2023). The ANOVA and Tukey HSD tests were used to identify differences between genotype (A1/A1, A1/A2, A2/A2) for longevity, reproductive, and production traits within each breed. *Post hoc* testing was conducted separately for each ewe breed.

## 3 Results

### 3.1 Statistical analyses of longevity, reproductive, and production traits

**FIGURE 1:**
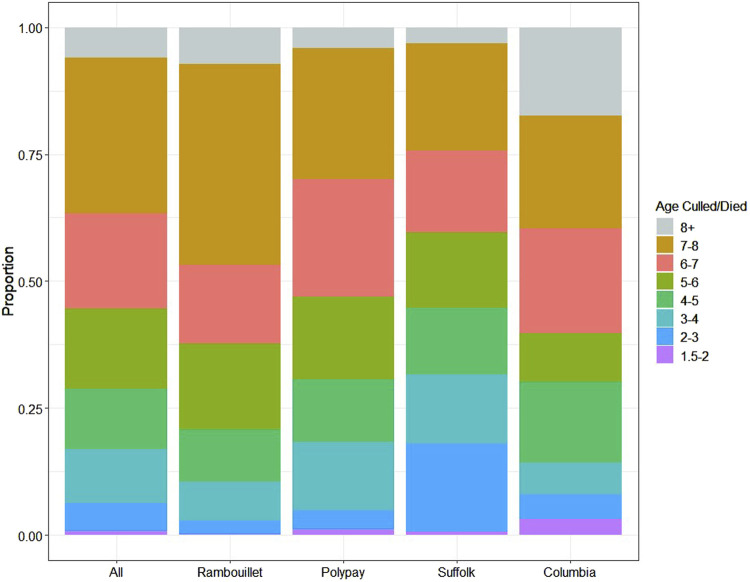
The age at which ewes left USSES management by death or culling. The age categories 1.5–2, 2–3, 3–4, 4–5, 5–6, 6–7, 7-8, and 8+ years were used to categorize the proportion of ewes leaving the flock through death/culling.

Ewes were born between 1999 and 2016 (Supplementary Figure S2) and lambed from 2001 to 2021 (Supplementary Figure S3). Individual longevity_1.5_ for study ewes ranged from 1.59 to 9.08 years, with an arithmetic mean of 6.00 ± 1.71 years (Table 1). The greatest proportion of ewes were culled or died at 7–8 years of age (30.53%), 6–7 years of age (18.76%), and 5–6 years of age (15.89%) (Figure 1; Supplementary Figure S4).

**TABLE 1:** Descriptive statistics for longevity, reproductive and production traits. Statistics are presented as the average ± standard deviation. R, Rambouillet; P, Polypay; S, Suffolk; All, all breeds.

		Longevity_1.5_ (Years)	Longevity_3.35_ (Years)	Parity	LT No. Lambs born	LT No. Lambs born alive	LT No. Lambs weaned	LT Wt. Weaned (kg)
R	AVG +/- SD	6.34 ± 1.56	6.53 ± 1.36	5.21 ± 1.69	9.64 ± 3.68	9.17 ± 3.63	7.47 ± 3.14	260.89 ± 106.91
	Min	1.70	3.46	0.00	0.00	0.00	0.00	0.00
	Max	9.08	9.08	8.00	19.00	17.00	15.00	499.86
	N	433	480	480	480	480	480	480
P	AVG +/- SD	5.87 ± 1.64	6.10 ± 1.43	5.03 ± 1.74	10.65 ± 4.36	9.89 ± 4.30	7.79 ± 3.52	271.73 ± 120.43
	Min	1.59	3.40	0.00	0.00	0.00	0.00	0.00
	Max	8.55	8.55	8.00	22.00	21.00	16.00	549.75
	N	388	404	404	404	404	404	404
S	AVG +/- SD	5.31 ± 1.91	6.01 ± 1.46	4.19 ± 2.01	7.14 ± 3.74	6.73 ± 3.58	5.13 ± 3.04	207.52 ± 125.20
	Min	1.59	3.47	0.00	0.00	0.00	0.00	0.00
	Max	8.57	8.57	8.00	15.00	15.00	13.00	481.26
	N	161	182	182	182	182	182	182
C	AVG +/- SD	6.14 ± 1.97	6.56 ± 1.55	5.13 ± 2.01	8.72 ± 4.34	8.38 ± 4.41	6.74 ± 3.65	253.90 ± 134.25
	Min	1.59	3.49	1.00	1.00	0.00	0.00	0.00
	Max	8.59	8.59	8.00	18.00	18.00	13.00	456.09
	N	63	64	64	64	64	64	64
All	AVG +/- SD	6.00 ± 1.71	6.30 ± 1.43	4.97 ± 1.81	9.55 ± 4.15	8.99 ± 4.06	7.17 ± 3.42	255.77 ± 118.42
	Min	1.59	3.40	0.00	0.00	0.00	0.00	0.00
	Max	9.08	9.08	8.00	22.00	21.00	16.00	549.75
	N	1,045	1,130	1,130	1,130	1,130	1,130	1,130

All of the Pearson correlation coefficients between ewe longevity_1.5_ and reproductive and production traits showed positive correlations, which ranged from *r* = 0.82 to *r* = 0.97 (Table 2). The thresholds described by Akoglu (2018) were used to determine the strength of each relationship. Longevity_1.5_ and parity (*r* = 0.93, *p*-value = 0.00E-00), LT number of lambs born (*r* = 0.82, *p*-value = 1.22E-249), LT number of lambs born alive (*r* = 0.82, *p*-value = 9.18E-255), LT number of lambs weaned (*r* = 0.84, *p*-value = 3.88E-278) and LT weight (kg) of lambs weaned (*r* = 0.86, *p*-value = 1.90E-304) were strongly correlated.

**TABLE 2:** Results of Pearson correlation testing of longevity, reproductive and production traits. The Pearson correlation coefficient (*r*) is displayed above the diagonal and correlation *p*-values are below the diagonal. This analysis was conducted with ewes from all breeds, with a total of 1,045 observations per trait.

	Longevity_1.5_	Parity	LT No. Lambs born	LT No. Lambs born alive	LT No. Lambs weaned	LT Wt. Weaned
Longevity_1.5_		0.93	0.82	0.82	0.84	0.86
Parity	0.00E+00		0.89	0.89	0.89	0.90
LT No. Lambs Born	1.22E-249	0.00E+00		0.97	0.88	0.84
LT No. Lambs Born Alive	9.18E-255	0.00E+00	0.00E+00		0.90	0.87
LT No. Lambs Weaned	3.88E-278	0.00E+00	0.00E+00	0.00E+00		0.97
LT Wt. Weaned	1.90E-304	0.00E+00	8.70E-285	2.13E-320	0.00E+00	

### 3.2 Genome-wide association study results

#### 3.2.1 Across-breed analyses

**FIGURE 2:**
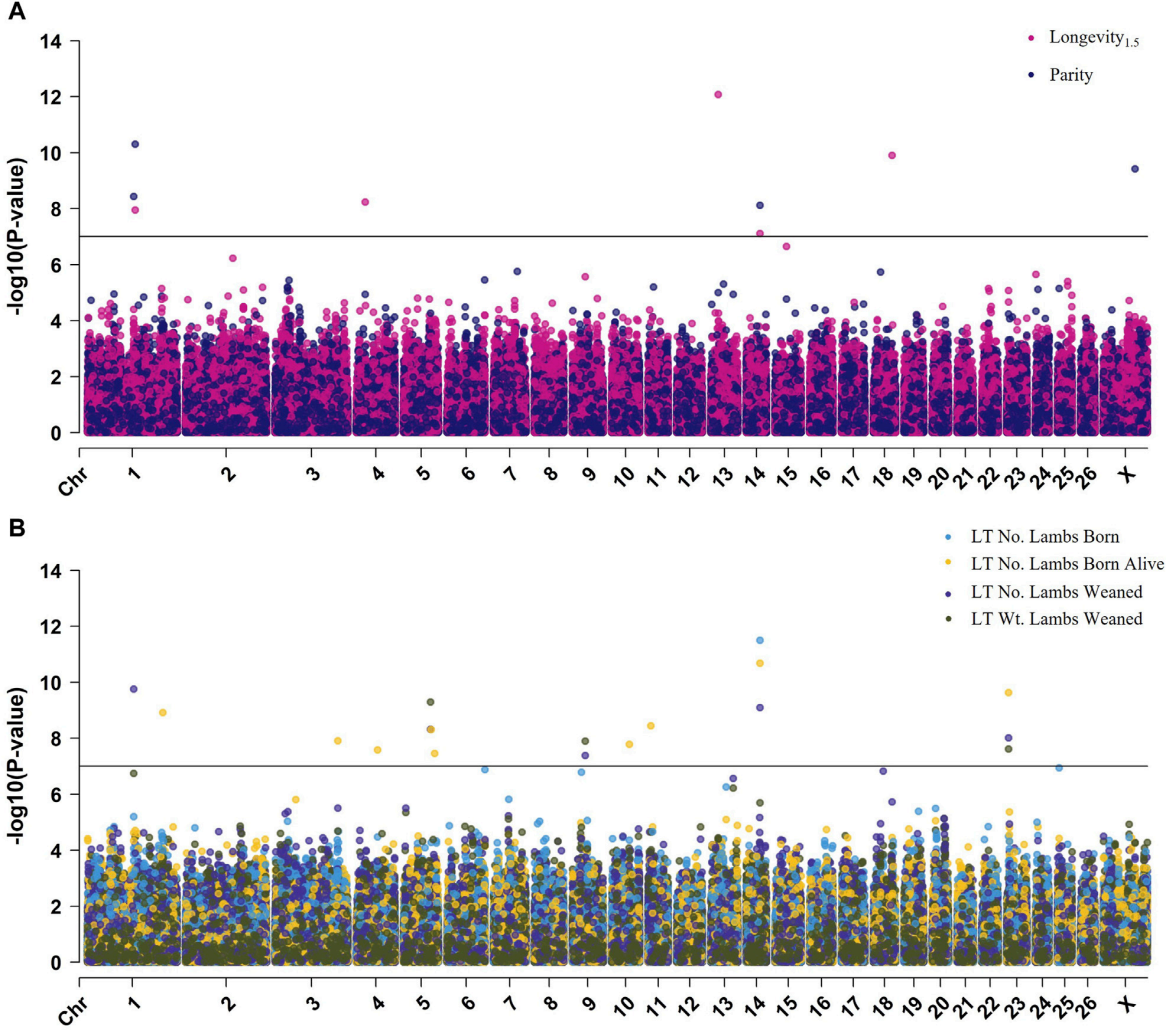
Multi-Manhattan plot of results from across-breed GWAS. **(A)** Results for longevity1.5 and parity **(B)** Results for LT number of lambs born, LT number of lambs born alive, LT number of lambs weaned, and LT weight of lambs weaned. The Bonferroni-adjusted *p*-value is used to represent genome-wide significance and is given by the horizontal black line (−log10 [*p*-value] = 7.00).

The GWAS for longevity_1.5_, parity, LT number of lambs born, LT number of lambs born alive, LT number of lambs weaned, and LT weight of lambs weaned identified 25 genome-wide significant SNPs; however, there were no significant results for longevity_3.35_ (Figure 2; Supplementary Figures S5, S6). Significant SNPs ranged in *p*-value from 2.7E-07 to 8.3E-13; the most significant SNP, rs411309094, located near the genes *COMMD3* and *BMI1*, was identified in the longevity_1.5_ GWAS. All GWAS showed adequate control of inflation as evaluated by both quantile-quantile plots and lambda genomic inflation factor statistics (Supplementary Figure S7).

The unadjusted *p*-value (*P*) and effect size (ES) were estimated by year (yr.), parity (ps.), number of lambs born, born alive, or weaned (no.), and weight of lambs weaned (kg). The average and standard deviation for each trait at the homozygous reference, homozygous alternate, and heterozygous genotype of significant SNPs are reported in Supplementary Table S1.

##### 3.2.1.1 GWAS for ewe longevity and parity

The GWAS conducted for ewe longevity_1.5_ identified six SNPs reaching genome-wide significance (Table 3). These SNPs included rs415130598 located on chromosome 1 (*p* = 1.1E-08; ES = +0.48 yr.), rs425691501 on chromosome 4 (*p* = 5.8E-09; ES = −0.4 yr.), rs411309094 near *COMMD3* and *BMI1* on chromosome 13 (*p* = 8.3E-13; ES = −0.43 yr.), rs160938219 within exon 4 of the gene *CEP89* on chromosome 14 (*p* = 7.7E-08; ES = +0.35 yr.), rs425305900 within *PLEKHA7* on chromosome 15 (*p* = 2.3E-07; ES = −0.41 yr.), and rs425390706 located near *FAM181A* and *ASB2* on chromosome 18 (*p* = 1.2E-10; ES = −0.56 yr.) (Table 4). The MAF of significant SNPs ranged from 0.13 to 0.44, with the most significant SNP, rs411309094, having a MAF of 0.42.

**TABLE 3:** Unadjusted *p*-values of significant SNPs identified by across-breed GWAS. Genome-wide significance is indicated by (*). Unadjusted *p*-values <9.99E-04 were considered to be trending and are included of the purposes of comparison of results between correlated traits.

rsID	Gene region (±50 kb)	Longevity_1.5_	Parity	LT No. Lambs Born	LT No. Lambs Born Alive	LT No. Lambs Weaned	LT Wt. Weaned
rs428984751	*ROBO2 ^*	4.0E-05	3.7E-09*	1.0E-04	--	--	--
rs430676331	*ROBO2 ^*	8.6E-05	--	6.3E-06	2.4E-05	1.8E-10*	1.8E-07*
rs415130598	--	1.1E-08*	5.0E-11*	9.8E-05	2.0E-05	1.0E-04	3.4E-05
rs413202908	*ARHGEF26 ^*	--	--	1.0E-04	1.2E-09*	7.0E-04	1.0E-04
rs414982594	*SLCO1A2 ^, IAPP*	--	--	3.0E-04	1.3E-08*	3.1E-06	2.1E-05
rs425691501	--	5.8E-09*	1.2E-05	--	--	3.0E-04	--
rs410933573	*MINDY4 ^*	--	--	3.4E-05	2.6E-08*	2.0E-04	--
rs429026455	*--*	9.3E-05	5.5E-05	--	--	4.8E-09*	5.1E-10*
rs423593519	*LOC106991202*	--	--	5.3E-05	5.0E-09*	--	--
rs409248660	*--*	--	--	--	3.5E-08*	--	--
rs402649933	*CTBP1*	6.6E-05	--	1.3E-07*	7.3E-05	4.2E-05	6.3E-05
rs411654200	*MTSS1 ^*	--	--	1.6E-07*	3.6E-05	9.3E-05	5.7E-05
rs407502103	--	4.0E-04	7.0E-04	4.0E-04	7.8E-05	4.1E-08*	1.3E-08*
rs430618341	--	--	--	4.4E-05	1.6E-08*	2.0E-04	1.0E-04
rs419881227	*BZRAP1, SUPT4H1, RNF43 ^*	1.0E-04	--	--	3.6E-09*	--	--
rs411309094	*COMMD3, BMI1, SPAG6*	8.3E-13*	1.0E-05	--	4.6E-05	9.0E-04	3.9E-05
rs411513398	--	--	1.2E-05	--	1.0E-04	2.7E-07*	6.1E-07
rs418424091	*CEP89 ^, FAAP24, RHPN2*	--	7.6E-09*	--	--	--	--
rs160938219	*CEP89 ^, FAAP24, RHPN2*	7.7E-08*	--	3.2E-12*	2.1E-11*	8.0E-10*	2.0E-06
rs425305900	*PLEKHA7 ^*	2.3E-07*	1.7E-05	--	--	9.0E-05	8.0E-04
rs400500577	*TMEM266 ^, ETFA*	--	--	2.0E-04	9.0E-04	1.5E-07*	6.7E-05
rs425390706	*FAM181A, ASB2*	1.2E-10*	--	--	3.5E-05	1.9E-06	6.9E-05
rs412663585	*NETO1*	8.4E-06	5.8E-05	3.2E-05	2.4E-10*	9.8E-09*	2.5E-08*
rs416904791	*DISC1 ^*	--	--	1.2E-07*	3.7E-05	2.0E-04	9.0E-04
rs414862599	*MBNL3 ^*	7.2E-05	3.8E-10*	--	--	2.0E-04	5.3E-05

The use of ( ^ ) after a gene name indicates that the SNP is located within the given gene, unmarked genes indicate that the SNP is within ±50 kb of the gene.

**TABLE 4:** Effect size and MAF data of significant SNPs identified by across-breed GWAS. The effect size of SNPs of genome-wide significance are indicated by (*).

rsID	Chr	Position	A2/A1	MAF	Longevity_1.5_	Parity	LT No. Lambs born	LT No. Lambs born alive	LT No. Lambs weaned	LT Wt. Weaned
rs428984751	1	141,894,037	T/C	0.34	−0.29	−0.39*	−0.62	--	--	--
rs430676331	1	141,937,849	C/T	0.13	0.40	--	1.07	0.97	1.18*	33.17*
rs415130598	1	146,419,527	T/C	0.13	0.48*	0.59*	0.90	0.96	0.74	28.23
rs413202908	1	231,514,827	T/C	0.38	--	--	−0.65	−0.87*	−0.47	−18.70
rs414982594	3	193,395,228	C/T	0.38	--	--	0.58	0.81*	0.62	20.28
rs425691501	4	27,551,098	A/G	0.44	−0.40*	−0.32	--	--	−0.51	--
rs410933573	4	65,449,013	G/A	0.4	--	--	−0.68	−0.81*	−0.50	--
rs429026455	5	83,023,268	C/T	0.07	−0.53	−0.47	--	--	−1.40*	−52.67*
rs423593519	5	84,861,135	G/A	0.06	--	--	1.18	1.76*	--	--
rs409248660	5	95,451,663	C/T	0.04	--	--	--	1.95*	--	--
rs402649933	6	116,182,916	C/T	0.46	0.27	--	0.70*	0.60	0.54	18.52
rs411654200	9	28,244,997	T/C	0.17	--	--	−1.01*	−0.83	−0.67	−24.62
rs407502103	9	40,083,118	C/A	0.05	0.57	0.55	1.36	1.45	1.57*	57.76*
rs430618341	10	54,397,285	A/G	0.34	--	--	0.71	0.84*	0.53	19.77
rs419881227	11	8,852,278	C/T	0.07	0.54	--	--	1.66*	--	--
rs411309094	13	22,467,143	A/G	0.42	−0.43*	−0.26	--	−0.61	−0.43	−18.89
rs411513398	13	69,037,516	C/A	0.14	--	0.36	--	0.86	0.84*	29.41
rs418424091	14	42,570,811	A/G	0.25	--	−0.39*	--	--	--	--
rs160938219	14	42,582,743	T/C	0.4	0.35*	--	1.10*	1.03*	0.79*	20.84
rs425305900	15	35,048,257	T/C	0.19	−0.41*	−0.33	--	--	−0.66	−20.22
rs400500577	18	30,472,991	C/T	0.14	--	--	0.93	0.80	0.97*	29.08
rs425390706	18	57,307,037	A/G	0.16	−0.56*	--	--	−0.88	−0.87	−25.71
rs412663585	23	5,140,645	T/C	0.23	0.38	0.34	0.82	1.14*	0.86*	29.89*
rs416904791	25	4,728,573	G/A	0.39	--	--	−0.80*	−0.64	−0.51	−15.87
rs414862599	X	97,108,285	A/G	0.46	0.26	0.37*	--	--	0.48	18.33

A1, minor allele; A2, major allele. The SNP positions are given according to the OARv4.0 References genome assembly.

Four SNPs were significantly associated with ewe parity at the genome-wide level (Table 3). The SNP rs428984751 positioned within *ROBO2* on chromosome 1 (*p* = 3.7E-09; ES = −0.39 ps.), rs415130598 within *MBNL3* on chromosome 1 (*p* = 5.0E-11; ES = +0.59 ps.), rs418424091 within *CEP89* on chromosome 14 (*p* = 7.6E-09; ES = −0.39 ps.), and rs414862599 on the X-chromosome (*p* = 3.8E-10; ES = +0.37 ps.). Of these, rs415130598 was also identified in GWAS for longevity_1.5_. The MAF of parity-associated SNPs ranged from 0.13 to 0.46 (Table 4).

##### 3.2.1.2 GWAS for reproductive and production traits

There were 17 SNPs identified as genome-wide significant in GWAS for LT number of lambs born, LT number of lambs born alive, LT number of lambs weaned, and LT weight of lambs weaned. Of these SNPs, five were identified in multiple LT reproductive traits, and 12 SNPs were identified in a single LT reproductive trait (Table 3). The most significant SNPs for LT traits were rs160938219 within *CEP89* on chromosome 14, rs430676331 within *ROBO2* on chromosome 1, and rs429026455 on chromosome 5. The SNP rs160938219 was the most significant for LT number of lambs born (*p* = 3.2E-12; ES = +1.1 no.) and number of lambs born alive (*p* = 2.1E-11; ES = +1.03 no.), rs430676331 was the most significant for LT number of lambs weaned (*p* = 1.8E-10; ES = +1.18 no.), and rs429026455 was the most significant for LT weight of lambs weaned (*p* = 5.1E-10; ES = −52.67 kg) (Table 4). The MAF of LT trait-associated SNPs ranged from 0.04 to 0.46.

##### 3.2.1.3 Significant SNPs identified across GWAS traits

As expected due to the strength of correlation between longevity, reproductive, and production traits, there was overlap between the results identified across multiple GWAS. In total, six SNPs reached significance for two or more traits (Table 3). As mentioned previously, rs415130598 was significant for both longevity_1.5_ and parity. The SNPs rs430676331, rs429026455, and rs407502103 were significant for LT number of lambs weaned as well as LT weight of lambs weaned. The SNP rs412663585 was significantly associated with the LT number of lambs born alive, LT number of lambs weaned, and LT weight of lambs weaned. The SNP rs160938219 was significant for longevity_1.5_, LT number of lambs born, LT number of lambs born alive, and the LT number of lambs weaned. Additionally, the SNPs rs428984751 and rs430676331 were identified in different traits but are both positioned within the first intron of the gene *ROBO2*.

#### 3.2.2 Post hoc testing of across-breed results

**FIGURE 3:**
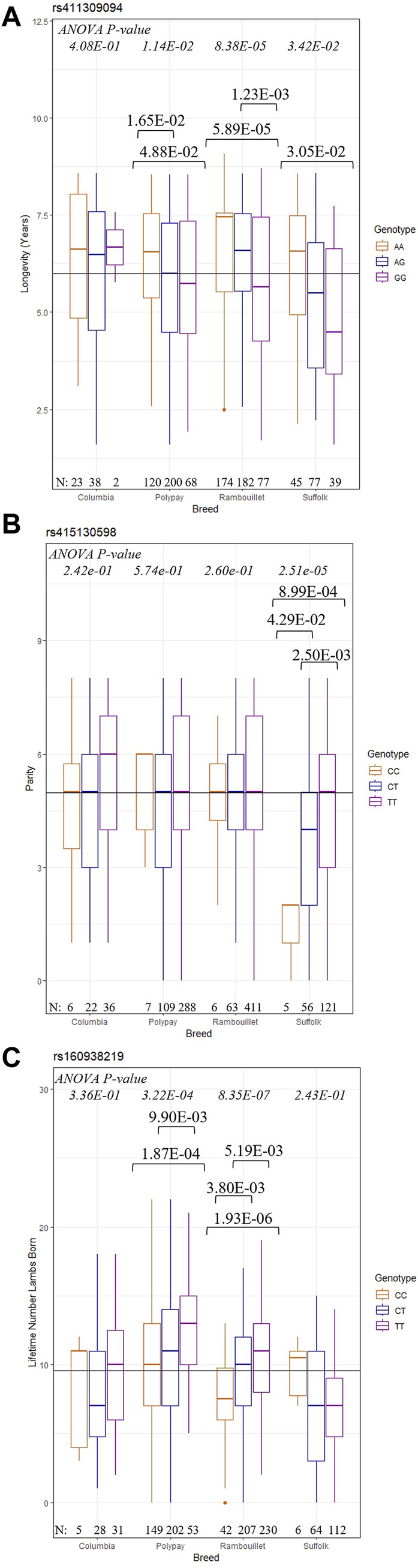
Results of *post hoc* testing of selected GWAS SNPs. The ANOVA *p*-values for each breed are reported at the top of the figure and Tukey HSD *p*-values are shown when significant. The number of individuals in each genotype/breed group are given at the bottom of figure. **(A)** Results of (longevity_1.5_ ∼ rs411309094) **(B)** Results of (parity ∼ rs415130598) **(C)** Results of (LT number of lambs born ∼ rs160938219).

To better interpret GWAS results, *post hoc* analyses with ANOVA and Tukey HSD tests were conducted to describe the relationship between significant SNPs and the longevity, reproductive, and production traits of each breed (Figure 3). At rs411309094, Rambouillet were found to have the most significant difference (*p* = 8.38E-05) between genotype and mean longevity_1.5_; Polypay and Suffolk ewes also had significant ANOVA tests (Figure 3A). For these breeds, ewes with the homozygous AA genotype had significantly greater mean longevity_1.5_ compared to ewes with the GG genotype. At rs415130598, Suffolk ewes with the CC genotype achieved a significantly lower mean parity than ewes with CT or TT genotypes (*p* = 2.51e-05) (Figure3B). The ANOVA results indicated that Rambouillet (*p* = 8.35E-07) and Polypay (*p* = 3.22E-04) ewes carrying the genotype TT for the SNP rs160938219 showed the highest LT number of lambs born, and ewes carrying the CC genotype showed the lowest LT number of lambs born (Figure 3C). The ANOVA and Tukey HSD results for the most significant SNP of each trait are reported in Supplementary Material (Supplementary Figures S8–S11).

#### 3.2.3 Within-breed analyses

**FIGURE 4:**
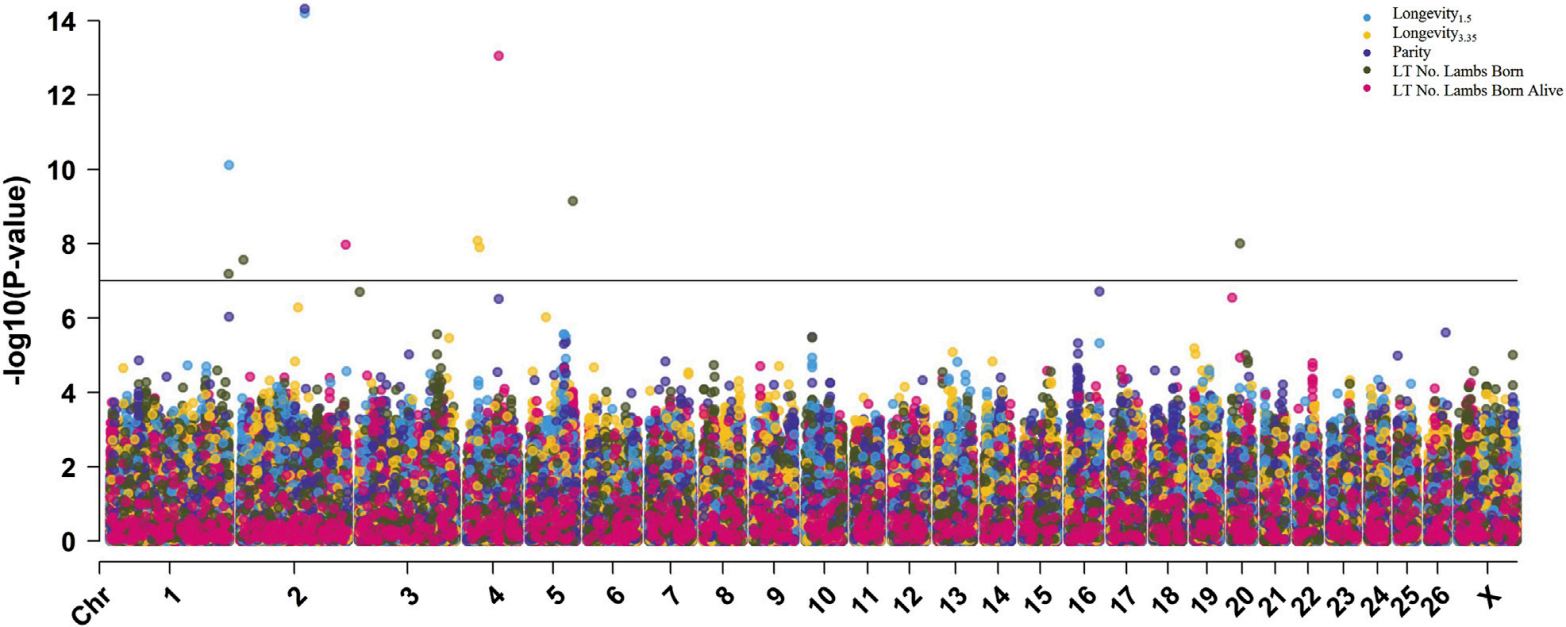
Multi-Manhattan plot of results from within-breed GWAS for Rambouillet ewes. The Bonferroni genome-wide significance threshold (-log10 [*p*-value] = 7.00) is represented by the horizontal black line.

To further explore the effect of breed on genomic associations with longevity and related traits, within-breed GWAS were conducted with the Suffolk, Polypay, and Rambouillet ewes (Table 5). Genome-wide significant results identified one SNP for longevity_3.35_ in Suffolk (Supplementary Figure S12); five SNPs identified for longevity_1.5_, parity, and LT number of lambs weaned for Polypay (Supplementary Figure S13); and 13 SNPs identified for longevity_1.5_, longevity_3.35_, parity, LT number of lambs born, and LT number of lambs born alive for Rambouillet (Figure 4; Supplementary Figures S14, S15). There were no significant associations identified for LT weight of lambs weaned. The most significant result of the within-breed analyses was rs429525276 within the gene *ITGB6*, associated with longevity_1.5_ (*p* = 6.4E-15; ES = 2.98 yr.) and parity (*p* = 4.8E-15; ES = −2.79 ps.) in Rambouillet ewes (MAF of 0.01) (Table 6). The QQ plots for each breed GWAS are included as Supplementary Figures (Supplementary Figures S16–18).

**TABLE 5:** Unadjusted *p*-values of significant SNPs identified by within-breed GWAS. Genome-wide significance is indicated by (*). Unadjusted *p*-values <9.9E-04 were considered to be trending and are included of the purposes of comparison.

Breed	rsID	Gene region (±50 kb)	Longevity_1.5_	Longevity_3.35_	Parity	LT No. Lambs born	LT No. Lambs born alive	LT No. Lambs weaned
S	rs409633557	*CAMK2D ^*	--	4.4E-10*	--	--	--	--
P	rs407010539	*GSDMC*	--	--	--	--	--	9.50E-10*
	rs416199190	*ATP8A2 ^*	1.5E-07*	3.3E-05	4.2E-05	1.0E-04	5.0E-04	--
	rs429722419	*ASIC2 ^*	1.2E-10*	2.8E-05	1.6E-09*	3.4E-06	7.2E-06	8.80E-11*
	rs411306494	*--*	--	--	6.0E-04	5.0E-04	4.0E-04	1.50E-08*
	rs413161211	*ANOS1 ^*	--	4.4E-05	3.1E-09*	7.0E-04	2.0E-04	--
R	rs415397995	*KCNH8 ^*	--	--	--	6.5E-08*	--	6.0E-04
	rs422040290	*KCNH8 ^*	7.7E-11*	9.0E-04	9.4E-07	--	--	--
	rs425779963	*ASTN2 ^ , TRIM32*	--	--	--	2.7E-08*	--	--
	rs429525276	*RBMS1, ITGB6 ^*	6.4E-15*	--	4.8E-15*	--	--	3.4E-05
	rs421700301	*ALPL ^*	--	--	--	--	1.1E-08*	--
	rs413643460	*OLFM1*	--	--	5.0E-04	2.0E-07*	--	2.0E-04
	rs410745921	*LOC105612556*	--	8.3E-09*	8.0E-04	--	--	2.4E-05
	rs422298481	*ABCB5*	--	1.3E-08*	--	--	--	7.0E-04
	rs400230536	*ZNF804B ^*	7.0E-04	2.0E-04	3.1E-07*	1.0E-04	8.9E-14*	6.2E-05
	rs403074344	--	--	--	2.0E-04	7.1E-10*	2.0E-04	1.5E-05
	rs418522450	--	4.7E-06	9.0E-04	2.0E-07*	--	8.0E-04	4.0E-04
	rs404678780	*PRIM2 ^*	--	--	--	1.0E-04	2.8E-07*	2.5E-05
	rs407236254	*LOC106990470 (PPIA), TNFRSF21 ^*	--	--	--	1.0E-08*	1.2E-05	2.0E-04

The use of ( ^ ) after a gene name indicates that the SNP is located within the given gene, unmarked genes indicate that the SNP is within ±50 kb of the gene. R, Rambouillet; P, Polypay; S, Suffolk.

**TABLE 6:** Effect size and MAF data of significant SNPs identified by within-breed GWAS. The effect size of SNPs of genome-wide significance are indicated by (*).

Breed	rsID	Chr	Position	A2/A1	MAF	Longevity_1.5_	Longevity_3.35_	Parity	LT No. Lambs born	LT No. Lambs born alive	LT No. Lambs weaned
S	rs409633557	6	12,163,186	C/T	0.27	--	1.07*	--	--	--	--
P	rs407010539	9	23,756,889	G/T	0.13	--	--	--	--	--	−1.88*
	rs416199190	10	33,625,826	G/A	0.13	−0.72*	−0.72	−0.69	−1.79	−1.65	--
	rs429722419	11	15,968,679	G/A	0.26	0.78*	0.62	0.77*	1.83	1.77	1.68*
	rs411306494	25	6,664,665	C/A	0.31	--	--	−0.43	−1.24	−1.26	−1.27*
	rs413161211	X	5,886,531	C/T	0.04	--	1.26	1.59*	2.85	3.1	--
R	rs415397995	1	274,248,112	A/G	0.24	--	--	--	1.28*	--	0.97
	rs422040290	1	274,343,886	A/C	0.34	−0.56*	−0.3	−0.4	--	--	--
	rs425779963	2	7,094,205	T/C	0.39	--	--	--	1.14*	--	--
	rs429525276	2	148,398,336	G/A	0.01	2.98*	--	2.79*	--	--	3.82
	rs421700301	2	244,125,440	C/T	0.45	--	--	--	--	−0.96*	--
	rs413643460	3	1,171,622	A/C	0.22	--	--	−0.39	−1.23*	--	−1.03
	rs410745921	4	24,201,492	T/C	0.08	--	0.7*	0.56	--	--	1.63
	rs422298481	4	29,352,185	G/A	0.35	--	−0.42*	--	--	--	−0.77
	rs400230536	4	73,315,205	C/T	0.43	0.3	0.3	0.43*	0.83	1.34*	0.91
	rs403074344	5	100,541,600	C/T	0.45	--	--	0.32	1.15*	0.69	0.92
	rs418522450	16	71,096,788	C/T	0.09	−0.63	−0.52	−0.76*	--	−1.15	−1.42
	rs404678780	20	2,688,106	T/C	0.14	--	--	--	1.2	1.3*	1.39
	rs407236254	20	20,384,085	C/T	0.21	--	--	--	−1.36*	−0.87	−1.01

A1, minor allele; A2, major allele. The SNP positions are given according to the OARv4.0 reference genome assembly. R, Rambouillet; P, Polypay; S, Suffolk.

## 4 Discussion

Ewe longevity is limited by culling and on-farm mortality (Flay et al., 2022). The reasons for ewes exiting the flock can vary by region, breed, and production management system (Annett et al., 2011; McLaren et al., 2020; Pelmus et al., 2020). A recent survey of 38 New Zealand sheep farmers revealed that nearly all responding producers (97%) cull mixed-aged ewes that fail to lamb (Ridler et al., 2023). Defects of the udder or teeth were common reasons for culling, with 82% of producers responding that they cull mixed-aged ewes with mastitis or ‘saggy’ and ‘blown-out’ udders, and 68% of producers reporting to cull ewes with missing, worn, excessively long, or wobbly teeth. Data from Norway, Ireland, and the UK revealed similar rationale for culling decisions made by commercial and research sheep farms (McLaren et al., 2020). Mastitis was cited as the most common reason for culling in Norway (19.9%), followed by udder problems (16.9%); ewe age and mastitis were the most common culling criteria in Ireland (20.9%, 13.5%); and in the UK, teeth problems constituted the main reason for culling (38.9%), followed by age (23.5%). A study with crossbred ewes in Northern Ireland identified failure to become pregnant (40.8%), udder problems (22.7%), and teeth condition (18.8%) as the main reasons for culling ewes (Annett et al., 2011). These reports indicate the importance of health (e.g., resistance to mastitis), soundness of the udder and teeth, and ability to become pregnant and maintain pregnancy as the major contributing factors to ewe longevity.

The current study utilized sheep data queried from previous projects. The distribution of age records described in these data are biased towards older ewes: only ewes greater than 1.5 years of age were considered, and more than 36% of study ewes were 7+ years of age at the time of their death or culling. Under typical practices, ewes would not be kept under USSES management past the age of 7. The main criterion used for ewe selection was litter weight of lambs weaned (Hanford et al., 2002; Hanford et al., 2005; Hanford et al., 2006), although ewes were also retained based on their involvement in ongoing research. The specific reasons for culling or death were not delineated, but ewes with the greatest longevity were expected to be largely unaffected by poor health or lack of productivity. With this unique dataset, we were able to conduct a robust GWAS for overall longevity.

### 4.1 Genetic associations with ewe longevity

The significant SNPs associated with longevity_1.5_ and longevity_3.35_ in across- or within-breed GWAS were positioned within 13 regions of interest containing 16 characterized genes. Many of these genes have been previously identified for roles or associations with the immune system. The gene *ASB2* has been reported to be involved in migration of natural killer cells, promotion of the Th2 type immune response, and regulation of the NF-κB pathway through the NF-κB inhibitor IκBα (Spinner et al., 2019; Sartori et al., 2021; Shin et al., 2021). Interestingly, *BMI1* has also been linked with NF-κB through regulation of IκBα ubiquitination (Okuyama et al., 2018). Notably, two genes implicated by longevity GWAS results have described connections to the programmed cell death protein 1 (PD-1) pathway, which has roles in response to infection and immune homeostasis (Sharpe and Paulken, 2018). Expression of the gene *RBMS1* has been correlated with PD1 ligand (PD-L1), and the receptor encoded by *ABCB5* has been shown to be co-expressed with PD-1 on dermal immunoregulatory cells (Schatton et al., 2015; Zhang et al., 2022). Additionally, the gene product of *Atp8a2* has been shown to be a Notch-regulated flippase important for regulation of intestinal intraepithelial lymphocytes in mice (Ishifune et al., 2019). Immune competency has been connected to both reproductive traits and health outcomes that influence longevity. Factors such as dystocia, fertility, lameness, parasite burden, and breech flystrike incidence could decrease longevity and correspondingly increase the number of mortalities during feedlot finishing in sheep or cattle (Banos et al., 2013; Hine et al., 2021; Hine et al., 2022). Taken together, these GWAS results broadly implicate the importance of immune pathways in ewe longevity and propose gene regions for further study in relation to the immune response and regulation of immune homeostasis in sheep.

The SNP rs411309094 was the most significant result of across-breed GWAS for longevity_1.5_. The presence of the G allele at this SNP was estimated to have an effect of −0.43 years. The region of interest defined by this SNP included genes *COMMD3*, *BMI1*, and *SPAG6*. As previously discussed, *BMI1* has ties to the immune system through pathways related to the transcription factor NF-κB. The COMMD family member COMM domain containing 3 (COMMD3) has been recently identified as a regulator of human epidermal growth factor receptor 2 (HER2) endosomal trafficking (Wang et al., 2023), and has potential roles in copper homeostasis in the progression of breast cancer (Hancock et al., 2023). Expression of the gene *SPAG6* is negatively correlated with prognosis for acute myeloid leukemia (AML) patients, and SPAG6 has been shown to interact with myosin 1D to increase expression of EGFR family within the context of AML (Mu et al., 2022). Mice deficient in *Spag6* had greater apoptosis and lower density of spiral ganglion neurons than wild-type mice, suggesting a potential role for *SPAG6* in auditory function (Li et al., 2017). While much of the existing research related to these genes has been conducted within the context of human cancers, there exists the potential for *COMMD3*, *BMI1*, and *SPAG6* to be involved in other biological processes important for ewe longevity. Further work is needed to explore the functions of these genes within the context of sheep health and survival.

The most significant SNP identified through within-breed GWAS was rs429525276, an intronic variant of the gene *ITGB6*. This SNP was significantly associated with both longevity_1.5_ and parity in Rambouillet ewes, and the G allele was estimated to have an effect of +2.98 years and +2.79 parities. The gene *ITGB6* has been associated with tooth enamel malformation in human patients (Poulter et al., 2014; Wang et al., 2014; Sriwattanapong et al., 2023), which is of particular interest within the context of ewe longevity, as ewes with worn or missing teeth are frequently culled. Sheep with no remaining teeth or poor teeth have decreased feed intake which reduces live weight gains and can negatively impact milk production, which in turn reduces the weaning weights of lambs (McGregor, 2011). Of further interest, a variant within *ITGB6* has been implicated in resistance or susceptibility to foot and mouth disease virus in zebu *versus* taurine cattle (Singh et al., 2015), and colorectal cancers have been shown to express ITGB6 to evade the antitumor cytotoxic T-cell response (Busenhart et al., 2022), suggesting important roles for *ITGB6* in promoting and maintaining health. The results of this GWAS propose *ITGB6* as a candidate for further study to elucidate the potential roles in tooth enamel formation and the immune response in sheep.

A previous study utilized USSES records of Columbia, Polypay, Rambouillet, and Targhee ewes born between 1950 and 2008 to estimate narrow sense heritability of longevity (Hanna et al., 2023). In across-breed analysis, heritability was estimated to be 0.16 ± 0.002; in within-breed analyses, estimates were 0.06 ± 0.02 for both Columbia and Polypay ewes, and 0.16 ± 0.02 for Rambouillet ewes. Although the breed composition and record timeframe used by Hanna et al. (2023) were not the same as the current study, these estimates can provide a basis of reference. According to these estimates, longevity is a lowly heritable trait, and the across- and within-breed GWAS results support the expectation that longevity can be extended through genetic selection.

### 4.2 Genetic associations with reproductive and production traits

Several of the genes implicated by these GWAS for reproductive and production traits have been previously associated with reproduction in sheep. Two significant SNPs identified for parity, LT number of lambs weaned, and LT weight of lambs weaned in across-breed GWAS are positioned within the genes *ANOS1* and *ROBO2*. Additionally, a SNP associated with LT number of lambs born alive in the Rambouillet GWAS is within the gene *ALPL*. The gene *ANOS1* has been previously found to be upregulated in the corpus luteum of highly prolific Finnsheep ewes compared to lowly prolific Texel ewes (Pokharel et al., 2020); similarly, *ALPL* was identified through differential gene expression of uterine tissue from polytocous and monotocous Small Tail Han sheep (La et al., 2019). The gene *ROBO2* has been previously implicated by multiple genome-wide studies, including GWAS for prolificacy in Pelibuey sheep, number of teats in a multi-breed analysis, and milk fat yield and milk protein yield in Valle del Belice sheep (Hernández-Montiel et al., 2020; Li et al., 2020; Mohammadi et al., 2022). Additionally, *ROBO2* has been shown to have dynamic gene and protein expression in fetal ovaries and is potentially related to follicle formation and maturation in sheep (Dickinson et al., 2010). The associations identified in the current GWAS highlight the potential importance of these genes in supporting reproductive performance in sheep.

### 4.3 Comparison of across- and within-breed GWAS

The diversity of GWAS results reported in this study may be explained in part by the algorithm used, as BLINK utilizes LD information to avoid redundancy in the significant markers reported for each trait (Supplementary Figure S19) (Huang et al., 2019). Differences in the sample sizes between across- and within-breed GWAS may have contributed to these results as well, as the within-breed analyses were more limited by sample number. Despite this, there is regional overlap of significant SNPs identified in the across- and within-breed analyses: specifically, rs425691501 associated with longevity_1.5_ in across-breed GWAS is located 1.80 Mb and 3.35 Mb from rs422298481 and rs410745921 associated with longevity_3.35_ in Rambouillet GWAS.

The effect sizes estimated for significant SNPs suggests that improvements can be made through genetic selection. Of note, rs429722419 had an effect size of +0.78 years for Polypay ewes and rs409633557 had an effect size of +1.07 years in Suffolk ewes. Such increases in longevity in Polypay and Suffolk breeds could lead to substantial improvements in profitability by limiting costs associated with replacing ewes. Reducing the frequency of the minor allele at SNP rs429026455 may improve overall weaning statistics, as it had an estimated effect size of −1.4 lambs for LT number of lambs weaned and −52.67 kg for LT weight of lambs weaned in across-breed GWAS. The MAF at this SNP is low (0.07), suggesting it could be further reduced relatively quickly. Additionally, SNP rs412663585 had high positive effect sizes for LT number of lambs born alive (+1.14 lambs), LT number of lambs weaned (+0.86 lambs), and LT weight of lambs weaned (+29.89 kg). Selection towards the major allele at this SNP could improve multiple reproductive and production traits at once, and therefore may be a candidate for inclusion in a balanced selection strategy.

## 5 Conclusion

Improving ewe longevity has the potential to provide economic benefits to the producer, as well as support favorable animal welfare and sustainable farming practices. However, longevity has historically been a difficult trait to select for, as it cannot be definitively determined until the end of a ewe’s productive life. The genetic associations identified in this study may improve ewe longevity if prioritized during the application of genomic selection. The genes implicated by GWAS results have described associations with reproduction, dentition, and immune function, and are proposed for further study to better elucidate the biological factors influencing ewe longevity in sheep managed under extensive rangeland systems.

## Data Availability

The original contributions presented in the study are included in the article/Supplementary Material, further inquiries can be directed to the corresponding authors.

## Supplementary material

Supplementary Table S1 The average and standard deviation statistics for each trait at the homozygous reference, homozygous alternate, and heterozygous genotype of significant SNPs for the across-breed and within-breed GWAS.

Supplementary Datasheet S1 The trait data used in this study and the genotype data for each significant SNP.

## Abbreviations

LT, lifetime; USSES, U.S. Sheep Experiment Station.
