# Supplementary material for: Genome-wide associations with longevity and reproductive traits in U.S. rangeland ewes
Source: Front Genet. 2024 May 27;15:1398123. doi: 10.3389/fgene.2024.1398123 (PMC11163081; doi:10.3389/fgene.2024.1398123)
Supplement: Supplementary file 1 [file Table2.DOCX]

Supplementary Material

# Supplementary Data

## Supplementary Figures


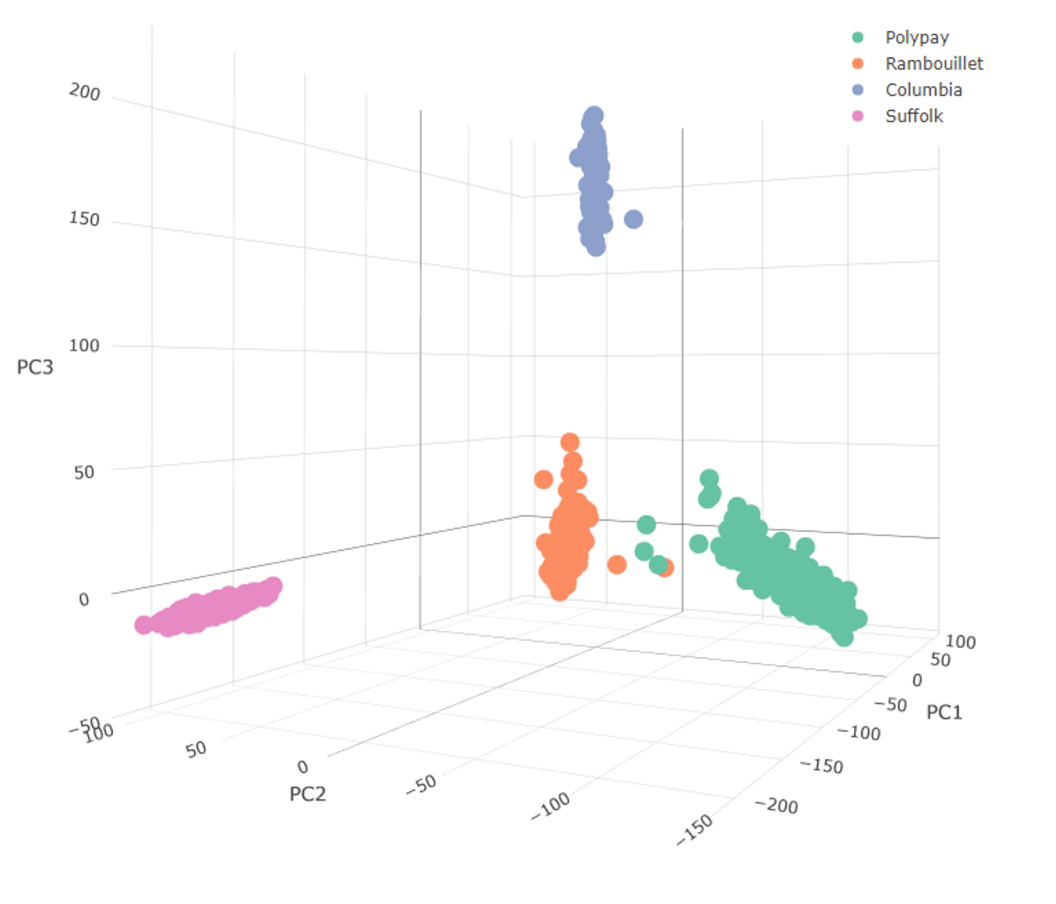


**Supplementary Figure 1.** Principal component analysis (PCA) with the first three PC. Individuals identified as Rambouillet are in orange, Polypay are in green, Suffolk are in pink, and Columbia are in blue.


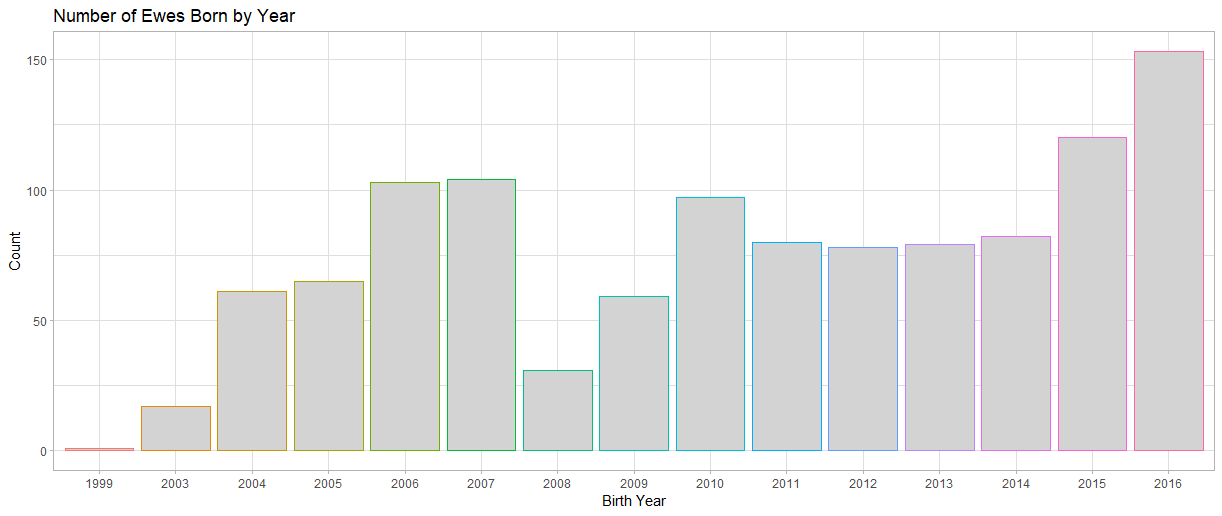


**Supplementary Figure 2.** Distribution of study ewes by year of birth.

**
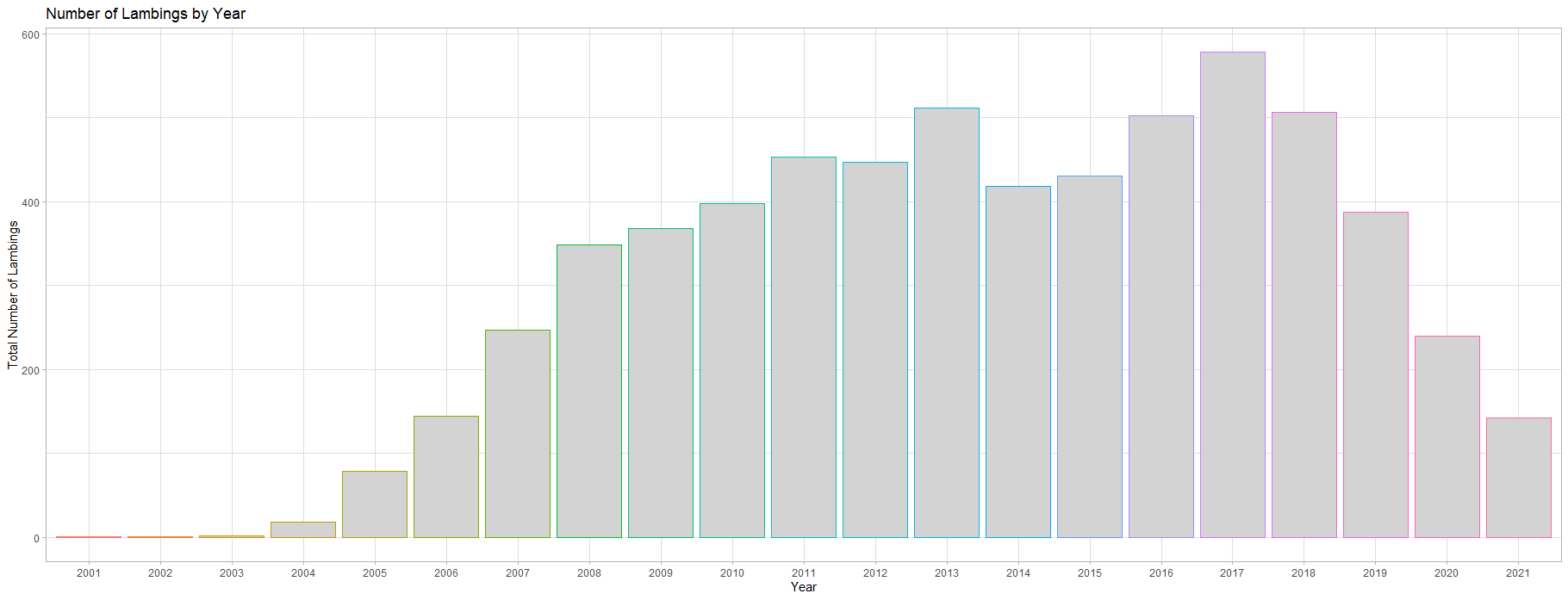
**

**Supplementary Figure 3.** Distribution of the number of lambings of study ewes by year.


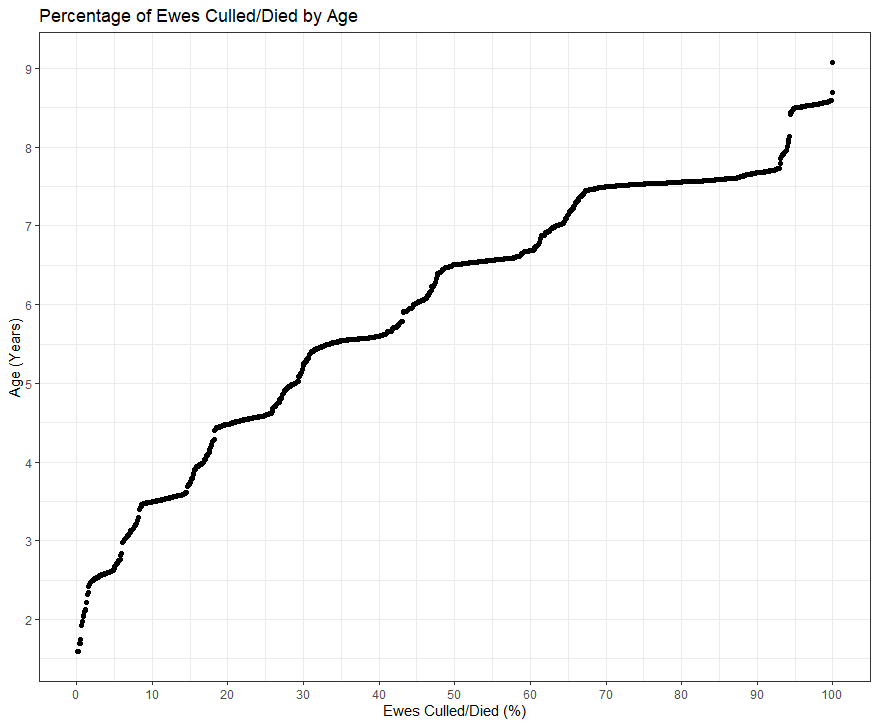


**Supplementary Figure 4.** Flock longevity visualized as the percentage of ewes that left the flock through culling or death by age in years.

**
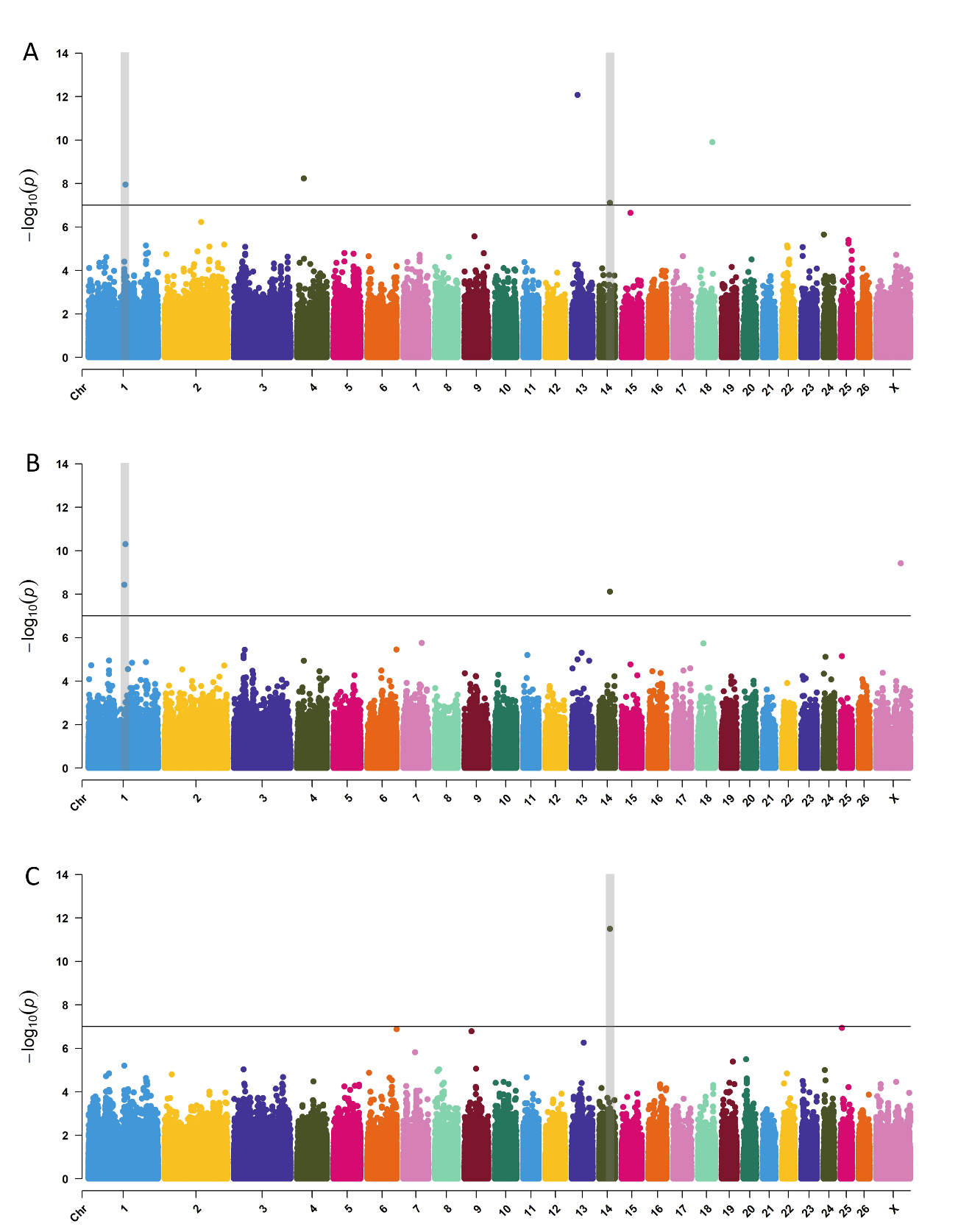
Supplementary Figure 5.** Results of across-breed GWAS by trait. (A) Results for Longevity_1.5_, (B) Results for parity, (C) Results for LT number of lambs born. In each panel, the gray boxes are used to identify markers that are significant in more than one trait.

**
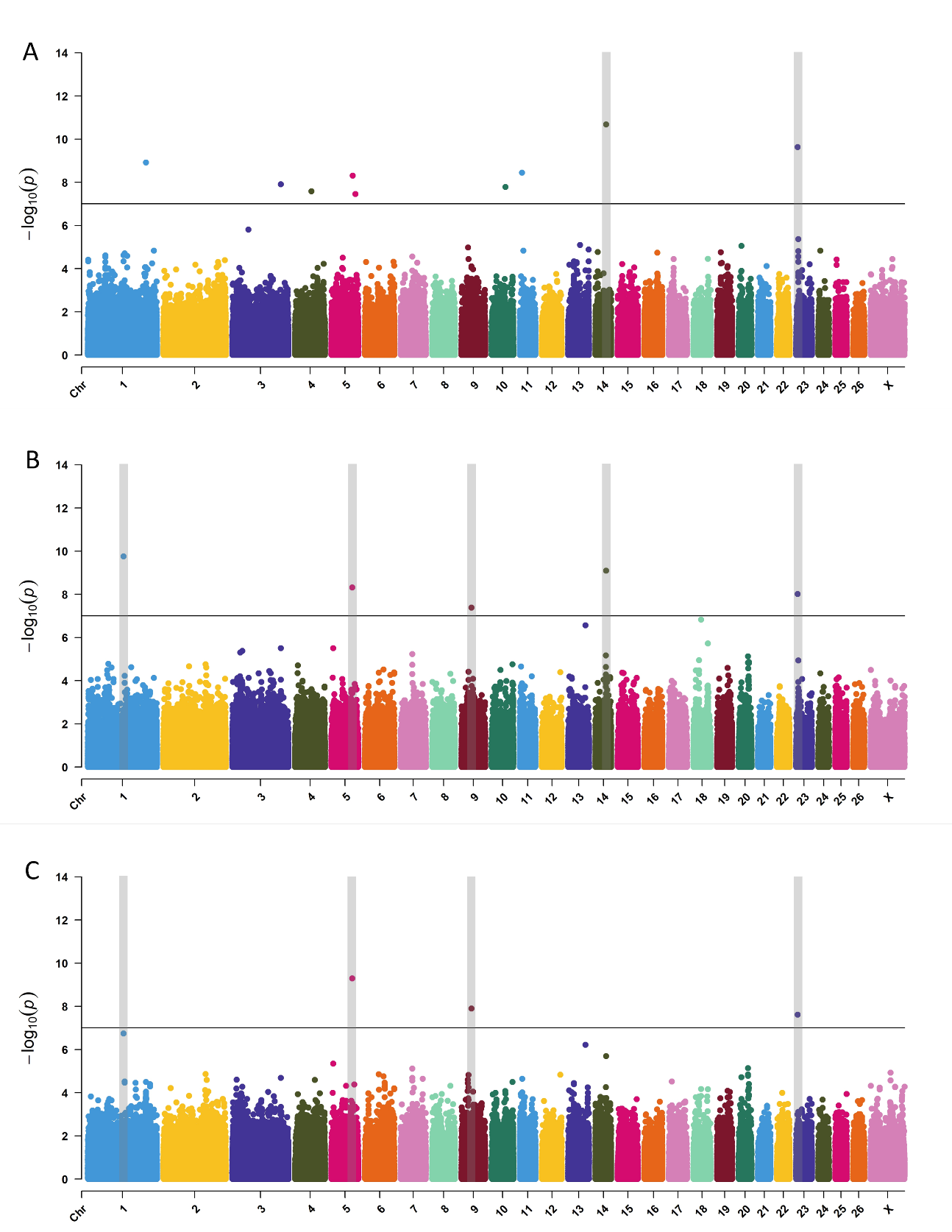
**

**Supplementary Figure 6.** Results of across-breed GWAS by trait. (A) Results for LT number of lambs born alive, (B) Results for LT number of lambs weaned, (C) Results for LT Wt. of lambs weaned. In each panel, the gray boxes are used to identify markers that are significant in more than one trait.

**
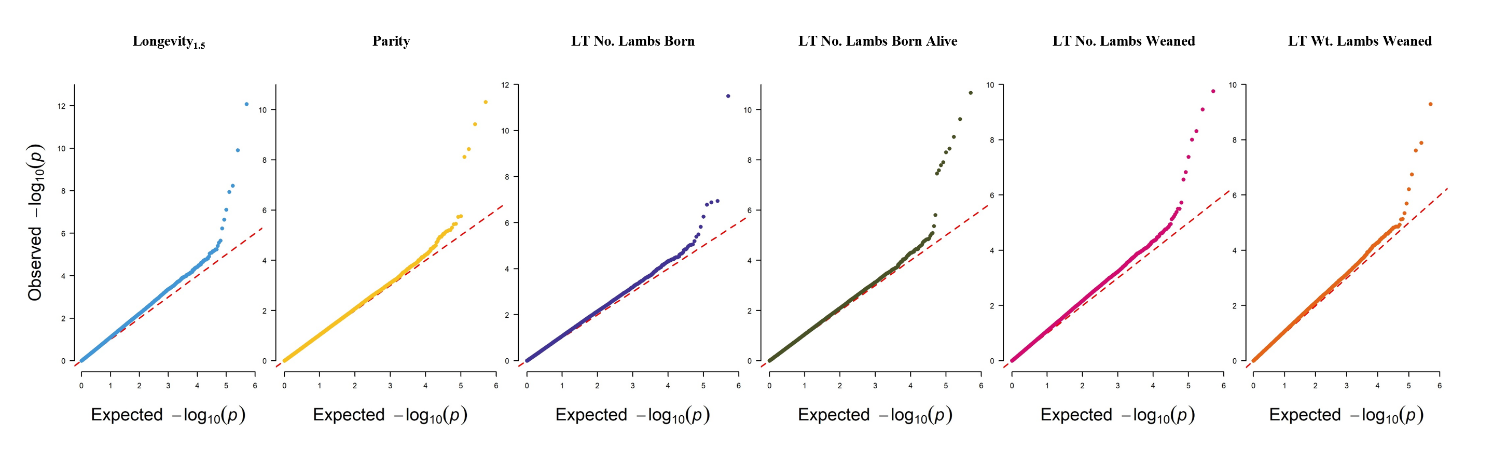
**

**Supplementary Figure 7.** Quantile-quantile (QQ) plots for across-breed GWAS. The lambda, genomic inflation factor, is displayed with each QQ plot.


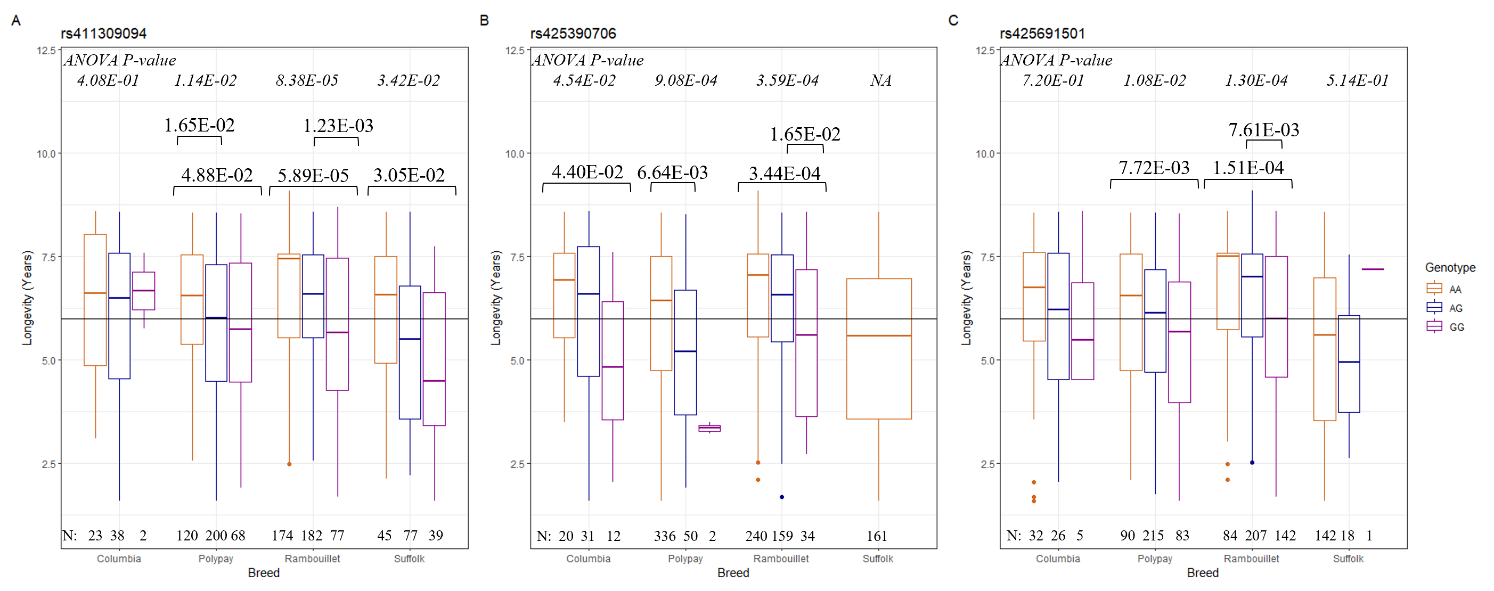


**Supplementary Figure 8.** Post hoc testing of the top three SNPs identified as significant for longevity_1.5_ in across-breed GWAS. (A) Results of (longevity_1.5_ ~ rs411309094), (B) Results of (longevity_1.5_ ~ rs425390706), (C) Results of (longevity_1.5_ ~ rs425691501).


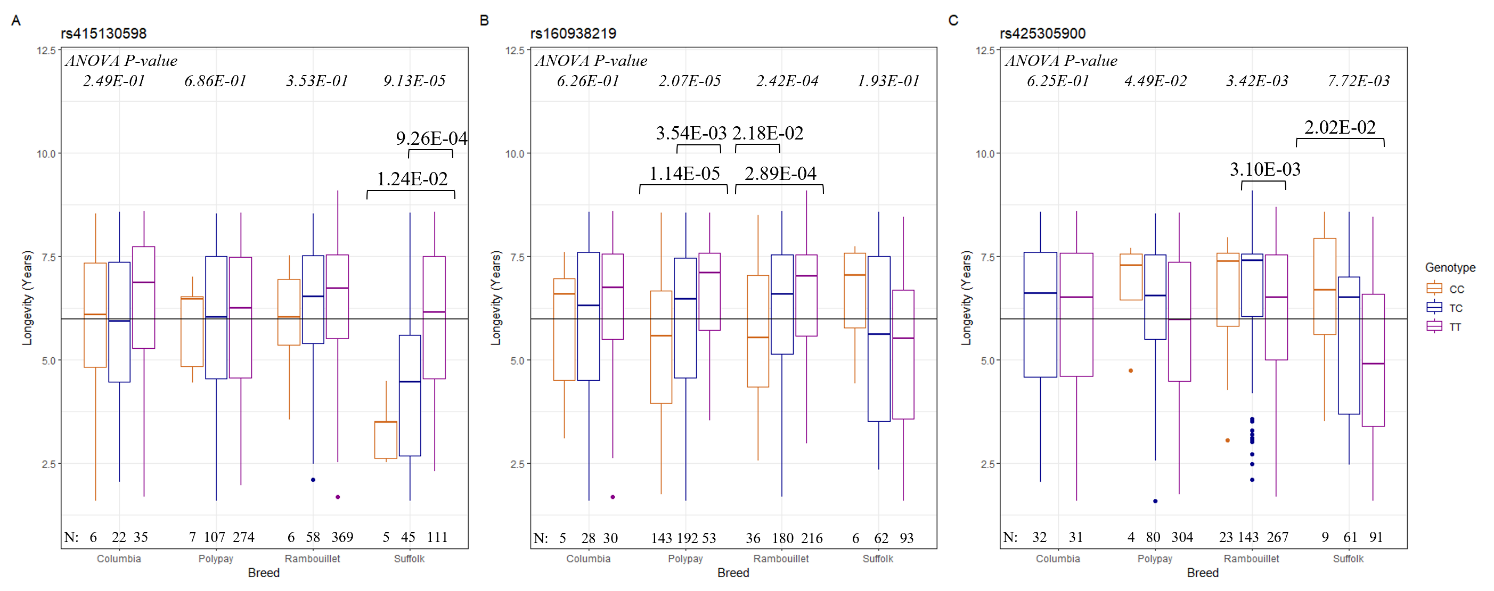


**Supplementary Figure 9.** Post hoc testing of the SNPs ranked fourth through sixth for significance with longevity_1.5_ in across-breed GWAS. (A) Results of (longevity_1.5_ ~ rs415130598), (B) Results of (longevity_1.5_ ~ rs160938219), (C) Results of (longevity ~ rs425305900).


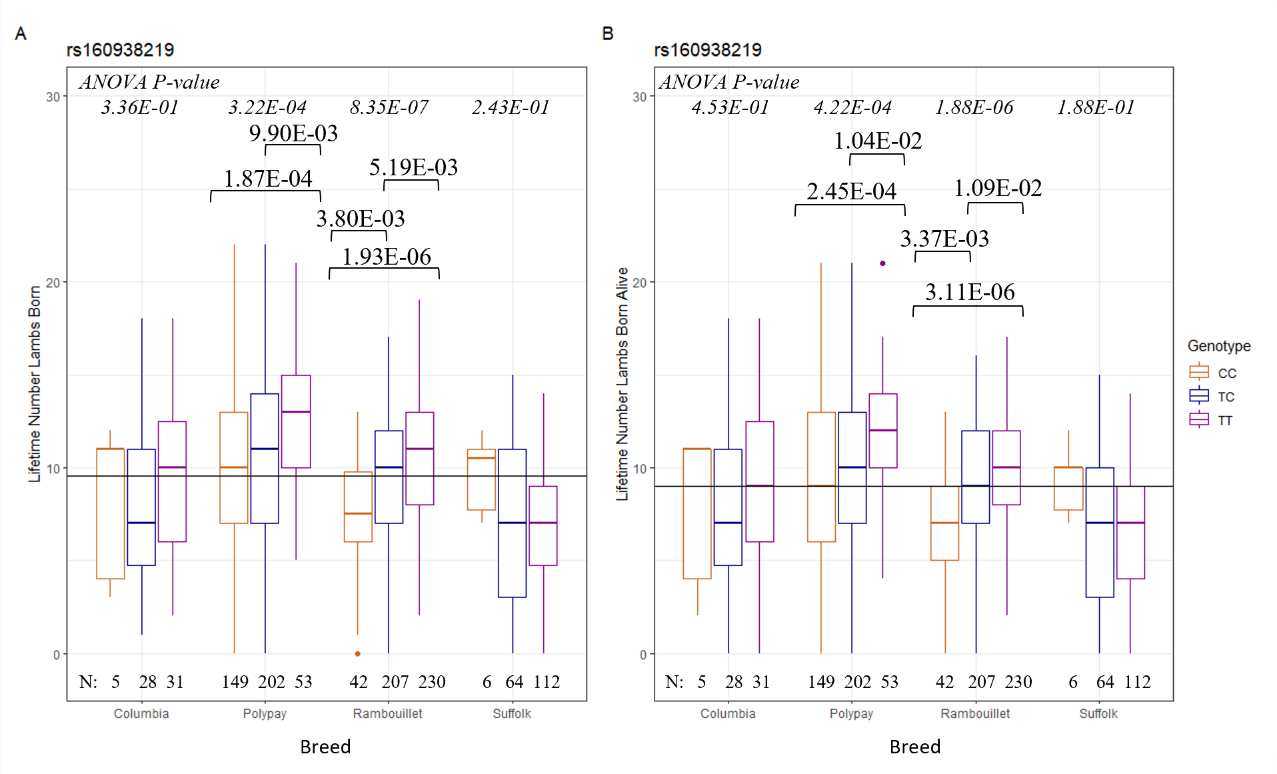


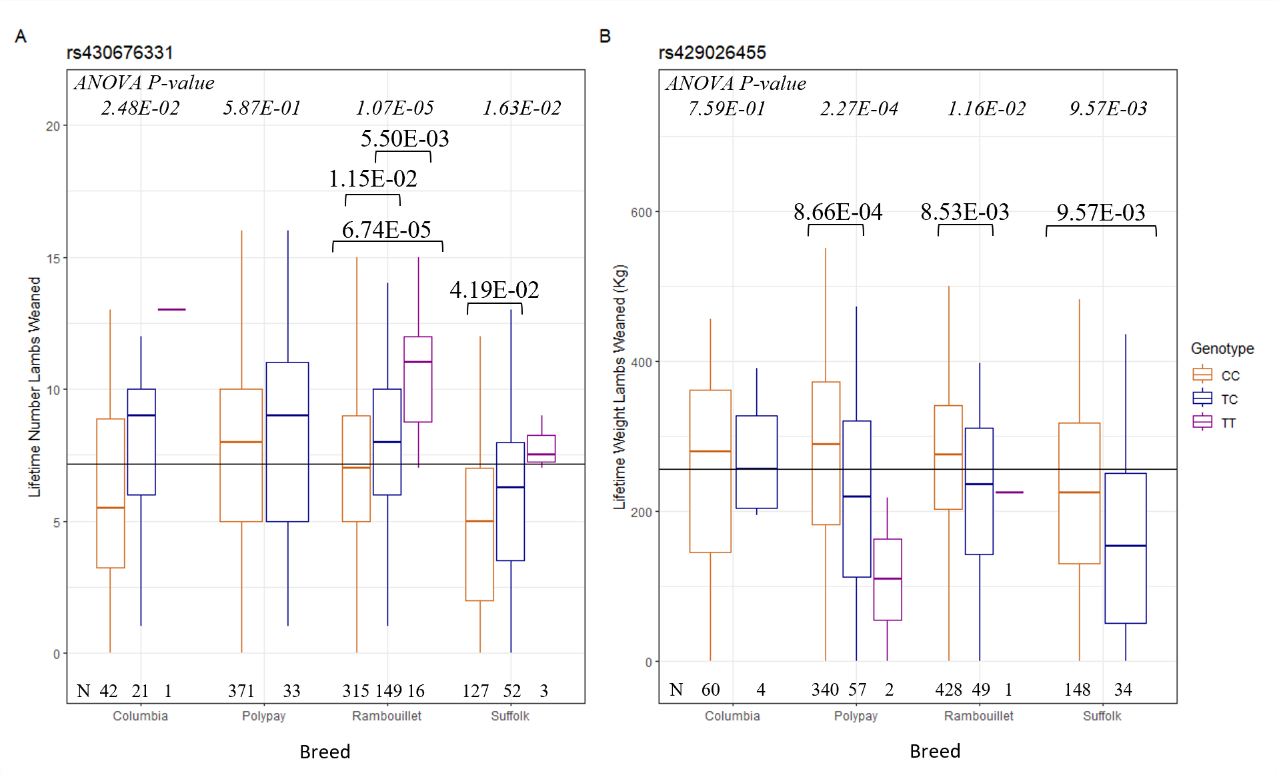


**Supplementary Figure 10.** Post hoc testing of the most significant SNP identified for lifetime reproduction traits in across-breed GWAS. (A) Results of (LT number of lambs born ~ rs160938219), (B) Results of (LT number of lambs born alive ~ rs160938219).

**Supplementary Figure 11.** Post hoc testing of significant SNPs identified for LT production traits in across-breed GWAS. (A) Results of (LT number of lambs weaned ~ rs430676331), (B) Results of (LT weight of lambs weaned ~ rs429026455).


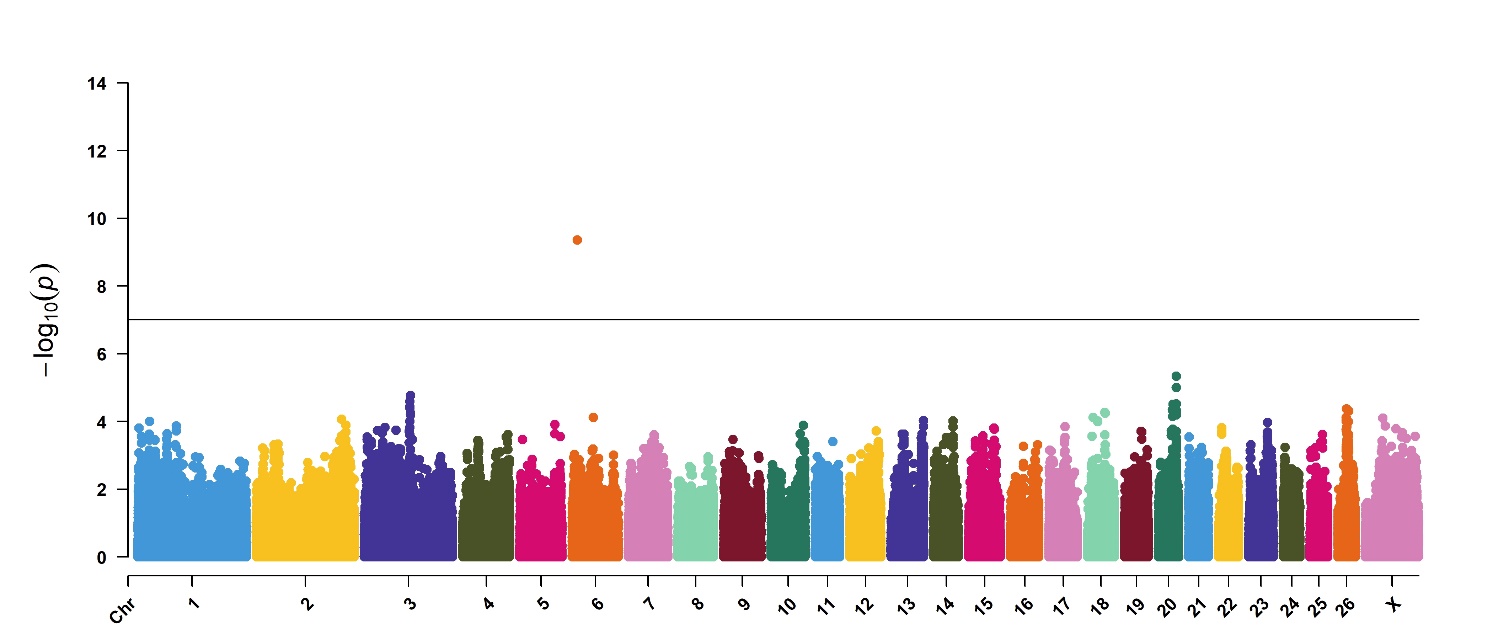
**Supplementary Figure 12.** Results of within-breed GWAS for Suffolk ewes. The black horizontal line represented the Bonferroni-adjusted *P*-value threshold (-log10 = 7.00).

**
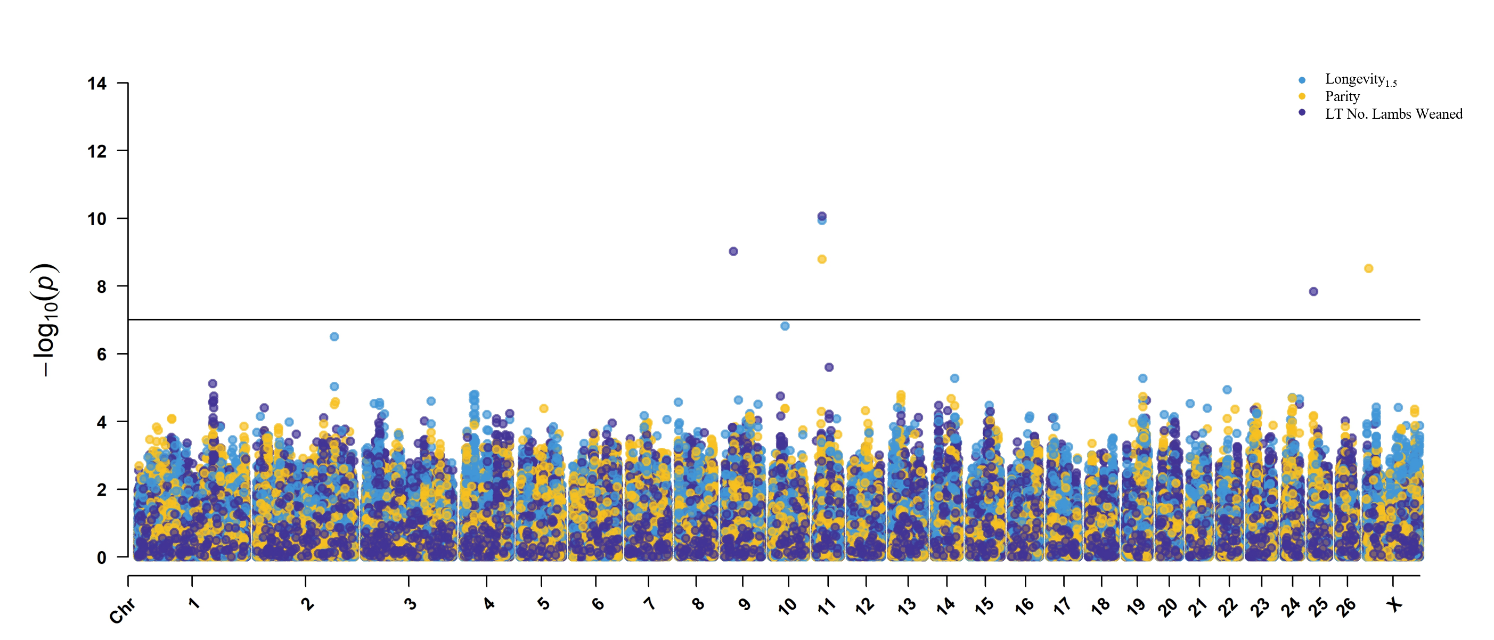
**

**Supplementary Figure 13.** Results of within-breed GWAS for Polypay ewes. The black horizontal line represented the Bonferroni-adjusted *P*-value threshold (-log10 = 7.00).


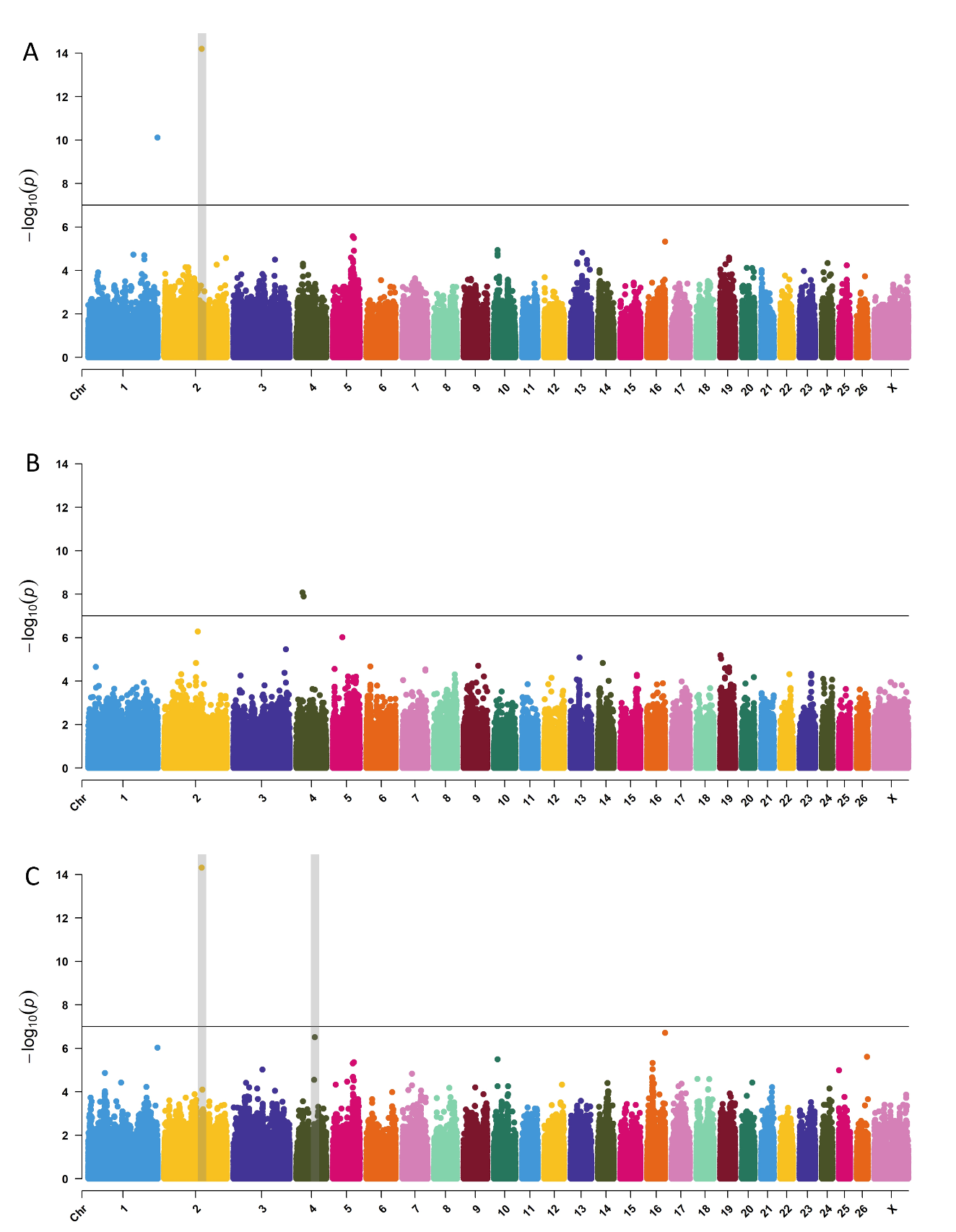


**Supplementary Figure 14.** Results of Rambouillet within-breed GWAS by trait. (A) Results for Longevity_1.5_, (B) Results Longevity_3.35_, (C) Results for parity. In each panel, the gray boxes are used to identify markers that are significant in more than one trait.


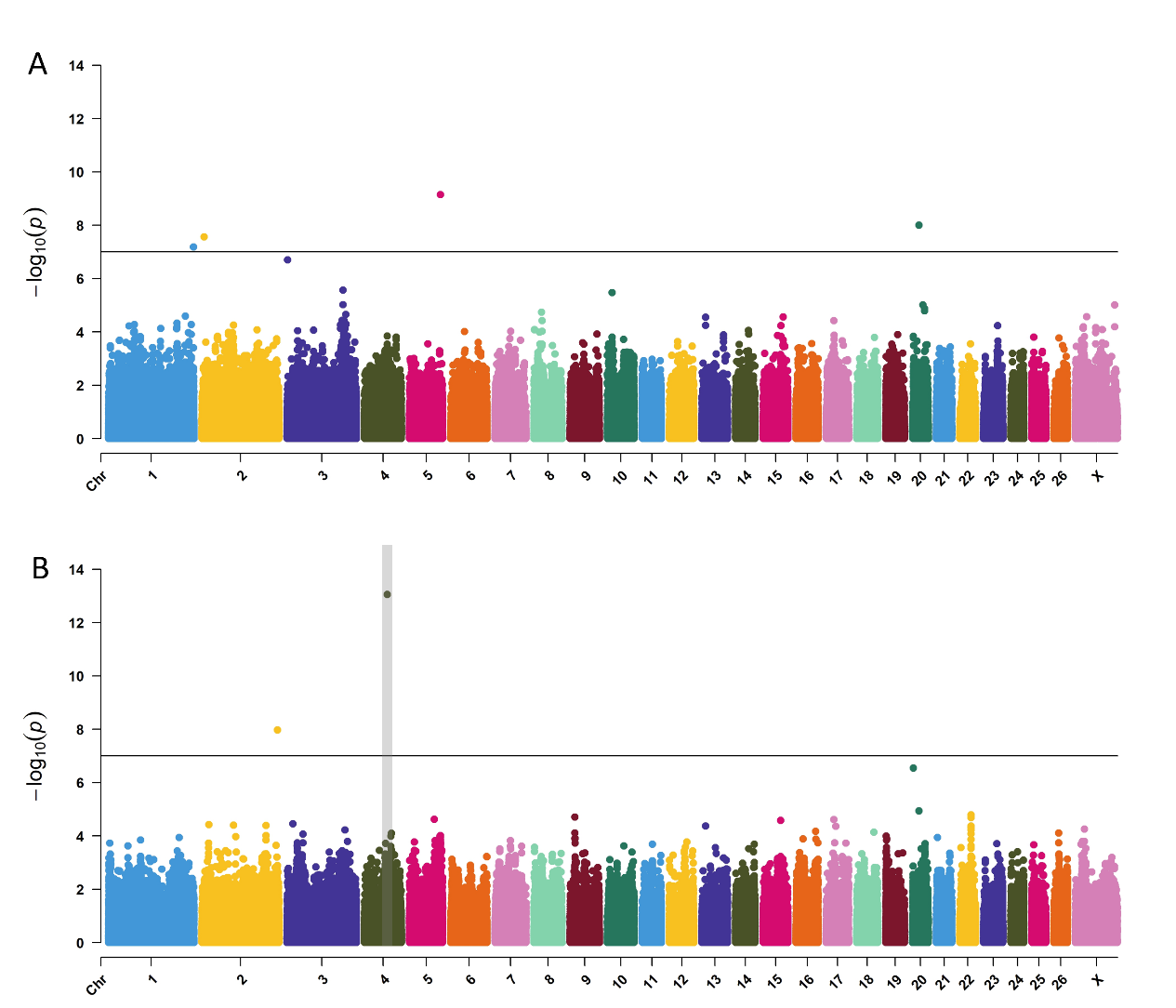


**Supplementary Figure 15.** Results of Rambouillet within-breed GWAS by trait. (A) Results for LT number of lambs born, (B) Results for LT number of lambs born alive. In each panel, the gray boxes are used to identify markers that are significant in more than one trait.


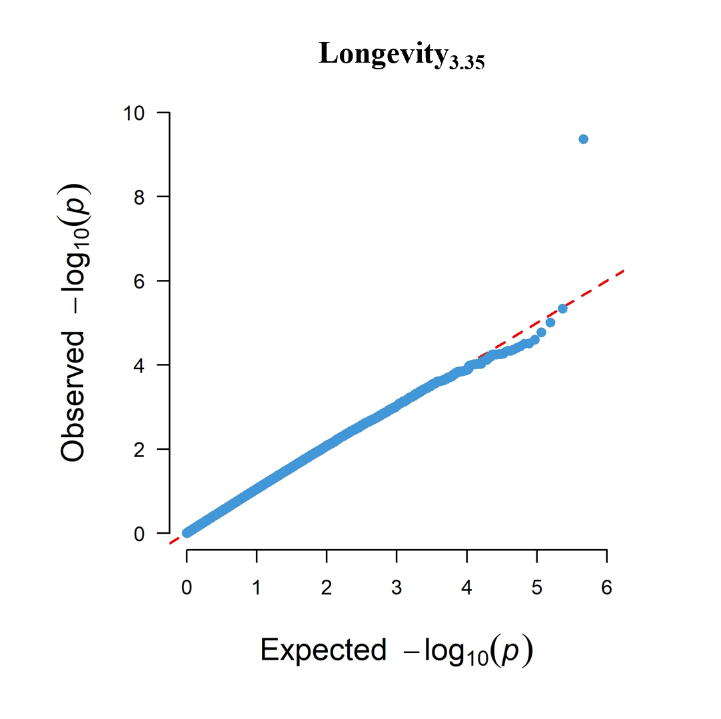


**Supplementary Figure 16**. QQ plots for Suffolk within-breed GWAS.


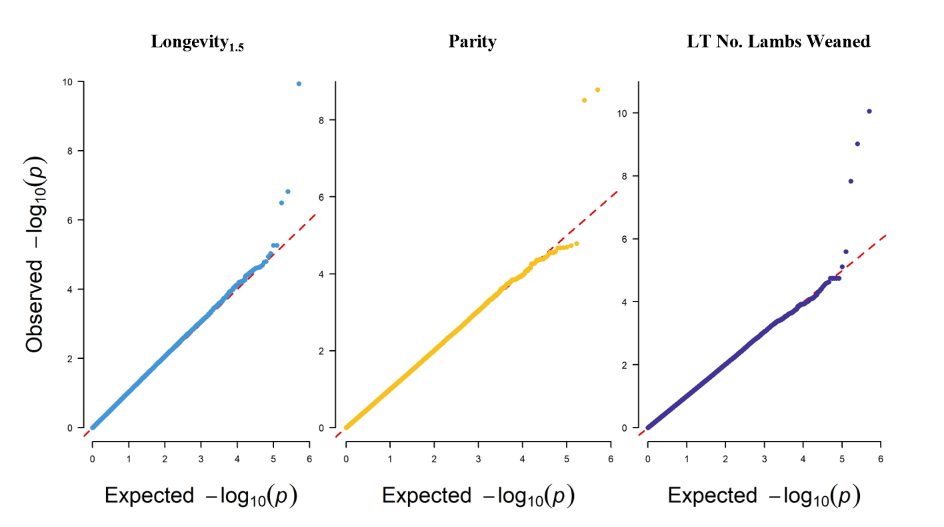


**Supplementary Figure 17.** QQ plots for Polypay within-breed GWAS.


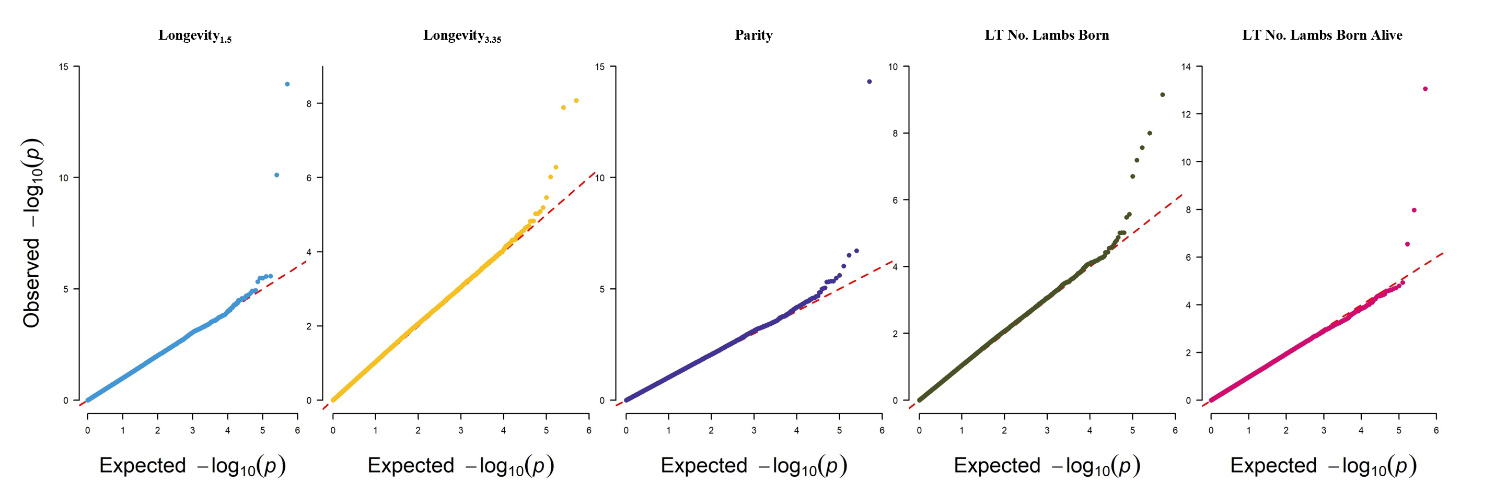
**Supplementary Figure 18**. QQ plots for Rambouillet within-breed GWAS.


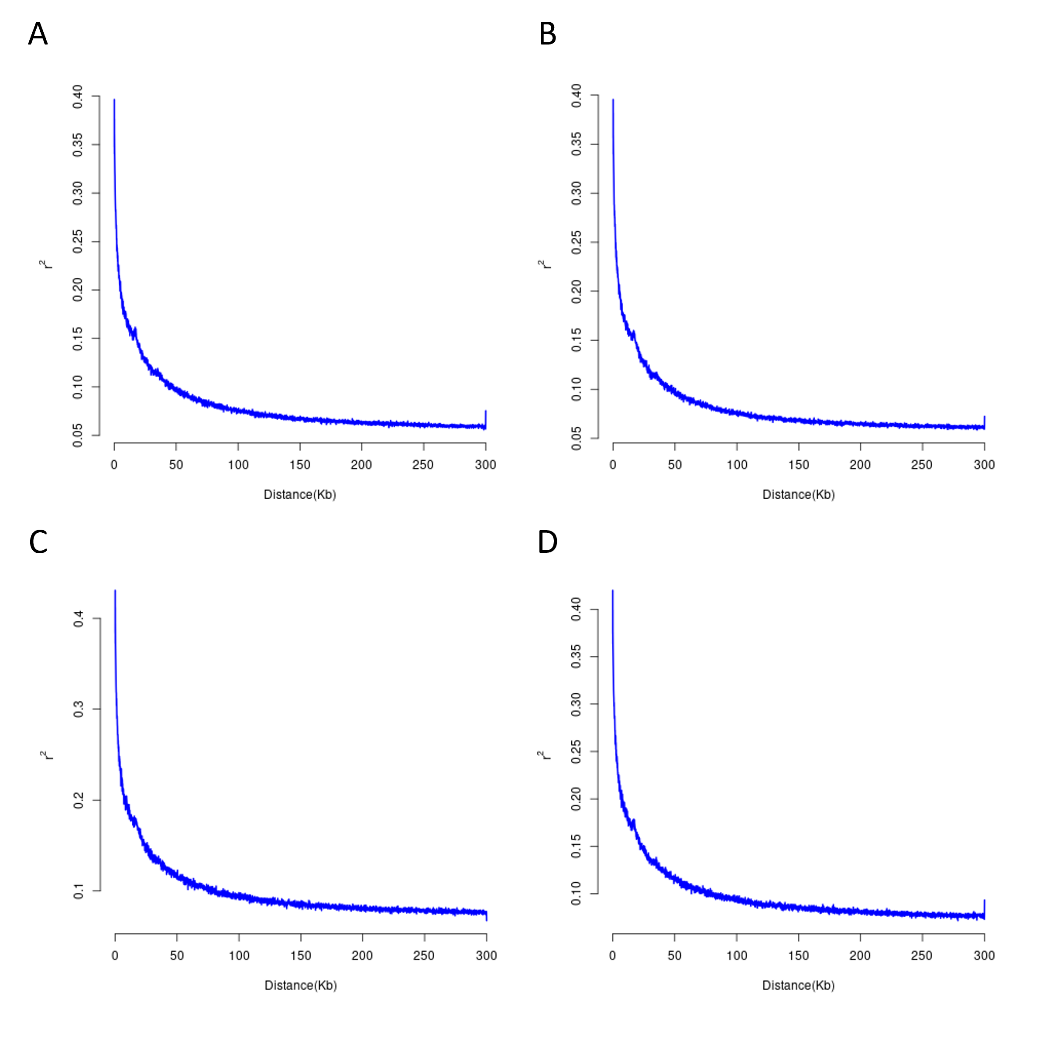


**Supplementary Figure 19.** Linkage disequilibrium (LD) decay over distance. The *r­­^2^* statistic was used to estimate LD. (A) LD decay in Rambouillet, (B) LD decay in Polypay, (C) LD decay in Suffolk, and (D) LD decay in Columbia breeds.
